# Palmitoylation of proteolipid protein M6 promotes tricellular junction assembly in epithelia of *Drosophila*

**DOI:** 10.1242/jcs.261916

**Published:** 2024-03-20

**Authors:** Raphael Schleutker, Stefan Luschnig

**Affiliations:** Institute of Integrative Cell Biology and Physiology, Cells in Motion (CiM) Interfaculty Centre, University of Münster, D-48149 Münster, Germany

**Keywords:** Epithelium, Tricellular junction, Cell vertex, Palmitoylation, *Drosophila*

## Abstract

Tricellular junctions (TCJs) seal epithelial cell vertices and are essential for tissue integrity and physiology, but how TCJs are assembled and maintained is poorly understood. In *Drosophila*, the transmembrane proteins Anakonda (Aka, also known as Bark), Gliotactin (Gli) and M6 organize occluding TCJs. Aka and M6 localize in an interdependent manner to vertices and act jointly to localize Gli, but how these proteins interact to assemble TCJs was not previously known. Here, we show that the proteolipid protein M6 physically interacts with Aka and with itself, and that M6 is palmitoylated on conserved juxta-membrane cysteine residues. This modification promotes vertex localization of M6 and binding to Aka, but not to itself, and becomes essential when TCJ protein levels are reduced. Abolishing M6 palmitoylation leads to delayed localization of M6 and Aka but does not affect the rate of TCJ growth or mobility of M6 or Aka. Our findings suggest that palmitoylation-dependent recruitment of Aka by M6 promotes initiation of TCJ assembly, whereas subsequent TCJ growth relies on different mechanisms that are independent of M6 palmitoylation.

## Teaser

**Highlighted Article:** The conserved fly proteolipid protein M6 is palmitoylated on cytoplasmic cysteine residues to promote localization at cell vertices, where M6 is required for assembly of tricellular occluding junctions.

## INTRODUCTION

Directional and selective transport across epithelia depends on occluding cell–cell junctions that seal the space between cells to restrict paracellular diffusion. Tight junctions (TJs) in vertebrates and septate junctions (SJs) in invertebrates play analogous roles as diffusion barriers, although they differ in their ultrastructure and molecular composition (reviewed in Higashi and Chiba, 2020). Junctions between two adjacent cells (bicellular junctions; BCJs) represent the most abundant intercellular contacts in epithelia. However, where three cells meet at cell vertices, BCJ strands are discontinuous and turn from having an orientation that is parallel to the apical cell surface to align with the central gap, which is flanked by three adjoining cells and sealed by specialized tricellular junctions (TCJs; Staehelin, 1973; Fristrom, 1982; Graf et al., 1982; Noirot-Timothée et al., 1982). TCJs are composed of a distinct set of proteins that mediate adhesive and occluding properties at cell vertices. In vertebrates, the tetraspan transmembrane (TM) protein tricellulin (also known as MARVELD2) (Ikenouchi et al., 2005) is recruited to tricellular tight junctions (tTJs) by the angulin family transmembrane protein lipolysis-stimulated lipoprotein receptor (angulin-1, also known as LSR; Masuda et al., 2011) and immunoglobulin-like domain-containing receptors 1 and 2 (ILDR1 and ILDR2; Higashi et al., 2013). TCJs play fundamental roles in epithelial barrier function, tissue integrity, cytoskeletal organization and mitotic spindle orientation (reviewed in Bosveld et al., 2018; Higashi and Chiba, 2020; Higashi and Miller, 2017). Mutations in tricellulin and ILDR1 cause deafness associated with degeneration of cochlear hair cells in mice and humans (Higashi et al., 2013; Riazuddin et al., 2006). Moreover, TCJs provide preferred routes for leukocyte extravasation from blood vessels (Burns et al., 1997; Sumagin and Sarelius, 2010) and are exploited by bacterial pathogens for breaching epithelial barriers (Papatheodorou et al., 2011; Sumitomo et al., 2016). However, despite the key functions of tTJs in epithelial biology and disease, the mechanisms underlying their assembly at cell vertices are only beginning to be understood.

Epithelia of invertebrates also display specialized tricellular occluding junctions. In *Drosophila*, the transmembrane proteins Anakonda (Aka, also known as Bark; Hildebrandt et al., 2015; Byri et al., 2015), Gliotactin (Gli; Schulte et al., 2003) and M6 (Zappia et al., 2011) organize tricellular septate junctions (tSJs) and are required for epithelial barrier function. Aka and M6 localize to TCJs in a mutually dependent fashion and act jointly to recruit Gli. Gli, in turn, is dispensable for targeting Aka and M6 to vertices, but is required for maintaining Aka and M6 localization, possibly by linking TCJ complexes to adjacent bicellular SJ strands (Esmangart de Bournonville and Le Borgne, 2020; Wittek et al., 2020).

A key open question is which specific features of cell vertices, and which corresponding cellular mechanisms, direct the assembly of TCJ complexes to this small portion of the plasma membrane. In *Drosophila*, Aka mediates homophilic cell adhesion with its triple-repeat-extracellular domain and is required on three adjoining cells for TCJ formation, suggesting that the extracellular domain of Aka recognizes vertex geometry or the presence of three adjacent plasma membranes (Byri et al., 2015). In addition, negative membrane curvature and a specific lipid composition at vertices might attract specific proteins through interactions mediated by special transmembrane or lipid-binding domains (Aimon et al., 2014). In vertebrates, angulin-1 is palmitoylated on cytoplasmic cysteine residues, and this post-translational modification is required, along with the extracellular domain, for its targeting to tTJs, possibly by mediating attraction to a cholesterol-enriched membrane domain at vertices (Oda et al., 2020).

S-palmitoylation is the covalent attachment of a C16 acyl chain to cytosolic cysteine residues of cytoplasmic or integral membrane proteins and is catalyzed by cytoplasmic palmitoyltransferases (reviewed in Chamberlain and Shipston, 2015). Unlike other lipid modifications, S-palmitoylation is reversible, and can hence regulate protein distribution or function in a switch-like manner. S-palmitoylation was shown to regulate partitioning of proteins into specific membrane domains, intracellular trafficking, protein– protein interactions, and activity of TM proteins (reviewed in Blaskovic et al., 2013).

In a search for molecular determinants that promote vertex localization of TCJ proteins, we found that the *Drosophila* proteolipid protein M6, like its vertebrate homolog GPM6a (Honda et al., 2017), is S-palmitoylated on a conserved cluster of juxtamembrane cysteine residues, whereas S-palmitoylation is not detectable on other known TCJ components in *Drosophila*. We show that S-palmitoylation promotes vertex localization of M6 and its direct interaction with Aka. S-palmitoylation of M6 is required for efficient initiation of TCJ assembly, but not for subsequent TCJ growth, thus revealing a distinct role of palmitoylation during an early step of TCJ assembly.

## RESULTS

### Three M6 isoforms are expressed in epithelia and localize to vertices

**Fig. 1.**
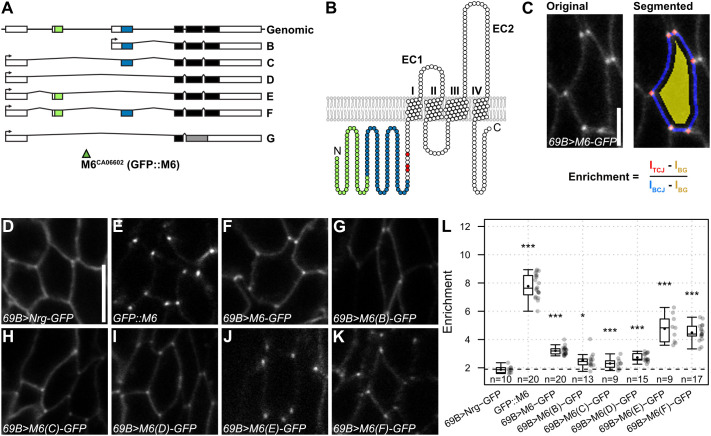
**All M6 protein isoforms localize to cell vertices.** (A) *M6* genomic locus (top) and transcripts encoding isoforms B–G (FlyBase). Exons shared by all isoforms are marked in black, isoform-specific exons are colored. Green triangle marks the M6::GFP (CA06602) protein trap insertion site. (B) Topology of M6 protein (isoform F). M6 isoforms comprise four TM domains, extra- and intra-cellular loops, and a variable N-terminus. Coloring corresponds to exons as in A. Palmitoylated cysteine residues are marked in red. (C) Analysis of vertex enrichment of M6–GFP fusion proteins. Example of a raw (left) and segmented (right) image of M6–GFP in an epidermal cell is shown. Vertex enrichment is calculated as the ratio of background-subtracted signals at TCJs and BCJs. (D–K) Distribution of Nrg::GFP (control; D), endogenous GFP::M6 (E) and UAS-M6–GFP (F–K) constructs expressed under control of *69B*-Gal4 in epidermis of living embryos (stage 15). M6-E and M6-F (J,K) show low signals and corresponding images were acquired with higher gain. (L) Quantification of vertex enrichment of Nrg–GFP (control), endogenous GFP::M6 and M6–GFP isoforms. Note that vertex enrichment of all M6 isoforms is significantly higher than that of Nrg–GFP. ***P≤0.001; *P≤0.05 (pairwise Wilcoxon rank-sum test with Holm correction). Each datapoint represents the mean of one embryo. Number of embryos (*n*) analyzed is indicated. Box plots show maximum and minimum observations within 1.5× interquartile range from the first and third quartile boundaries, upper and lower quartile, median (horizontal line) and mean (black dot). Scale bars: 2.5 µm (C); 5 µm (D–K).

To identify mechanisms that target M6 to vertices, we analyzed the subcellular distribution of M6 protein isoforms. The *M6* locus encodes six annotated protein isoforms (B through G; Fig. 1A; http://flybase.org/reports/FBgn0037092.htm; Thurmond et al., 2019), which differ in their N-terminal cytoplasmic domain. Isoforms B, C and D were detectable by immunoblotting in embryo lysates (Fig. S1A–C) and are expressed in ectodermal (isoforms C and D) and endodermal (isoform B) tissues (Fig. S1D–H).

We tagged each isoform C-terminally with GFP, drove expression of these constructs in the embryonic epidermis (Fig. 1D–K), and determined the enrichment of M6–GFP proteins at vertices as the ratio of signals at tricellular and bicellular contacts (Fig. 1C). This revealed that all M6 isoforms accumulated at vertices with mean enrichment factors of greater than 2.3-fold, whereas the bicellular SJ protein Neuroglian (Nrg–GFP; control) was enriched less than 2-fold (Fig. 1L). Notably, vertex enrichment varied between M6–GFP isoforms and was inversely proportional to overall signal intensity (Fig. S2), suggesting that saturation effects upon overexpression impede vertex enrichment. Consistent with this notion, endogenous GFP::M6^CA06602^ showed higher vertex enrichment (7.8-fold; Fig. 1E,L) than the individual overexpressed isoforms. We conclude that all M6 isoforms contain the element(s) sufficient for vertex localization.

### All M6 isoforms support TCJ formation

To test which M6 isoforms support TCJ formation, we expressed each isoform in epidermal stripes in *M6*-deficient [*M6^MB02608^/Df(3L)BSC419*] embryos and asked whether the M6 isoforms rescue TCJ localization of Aka and Gli, which are mislocalized along BCJs in the absence of M6 (Fig. S2). cDNAs of *M6* isoforms B, C, and D, as well as an intron-containing UAS-*M6* construct capable of producing all *M6* long-transcript isoforms, rescued TCJ localization of Aka and Gli (Fig. S2A–E). Isoforms M6-E and M6-F showed low expression levels and rescued TCJ localization of Gli, but only partially rescued Aka localization (Fig. S2F,G). However, in principle, all M6 isoforms can support TCJ formation.

### M6 protein is S-palmitoylated on a conserved cluster of cysteine residues

**Fig. 2.**
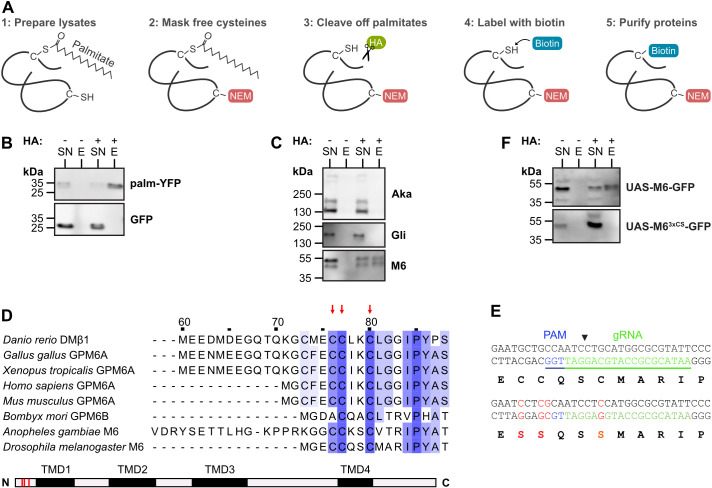
**M6 is palmitoylated on a conserved cluster of juxtamembrane cysteine residues.** (A) Principle of acyl-biotin-exchange (ABE) assay for detecting S-palmitoylation of proteins. Cell or embryo lysates are treated with N-ethylmaleimide (NEM) to mask free thiols, followed by hydroxylamine (HA) treatment to cleave off thioester-linked palmitate, thiol-specific biotinylation and purification of biotinylated proteins using streptavidin-coupled magnetic beads. (B) Lysates of embryos expressing palmitoylated YFP (palm-YFP) or unmodified GFP were subjected to the ABE assay. Eluate (E) and supernatant (SN) fractions after streptavidin purification were analyzed by immunoblotting and probed with anti-GFP antibodies. Presence or absence (control) of hydroxylamine (HA) treatment is indicated. Note that S-palmitoylation is detectable on palm-YFP, but not on unmodified GFP. (C) ABE assay to test for S-palmitoylation of the TCJ proteins Aka, Gli (Gli::YFP) and M6 (GFP::M6) in lysates of embryos (14 to 18 hAEL). Note that M6 (two bands corresponding to isoforms M6-B, M6-C and M6-D), but not Aka or Gli, is S-palmitoylated. (D) Multiple sequence alignment of N-terminus of *Drosophila melanogaster* M6 (isoform D) and M6 homologs from indicated species. Note conserved cluster of three cysteine residues (red arrows) in the juxtamembrane region shared by all *D. melanogaster* M6 isoforms. (E) CRISPR/Cas9-based genome editing approach to mutate the three clustered cysteine residues to serine residues (red) to generate palmitoylation-deficient M6 mutant (M6^3xCS^). Position of guide RNA (underlined), PAM and cut site (arrowhead) are indicated. (F) UAS-M6–GFP or UAS-M6^3xCS^–GFP were expressed in S2R+ cells and tested for palmitoylation. Note that M6^3xCS^ did not exhibit detectable palmitoylation. Two independent repeats were carried out for each ABE experiment shown in B, C and F.

We next asked which elements that are shared by all M6 isoforms mediate vertex localization. Because S-palmitoylation modulates membrane localization of the vertebrate M6 homolog GPM6a (Honda et al., 2017), we asked whether palmitoylation is involved in vertex localization of *Drosophila* M6. To test whether M6 is palmitoylated, we used an acyl-biotin-exchange (ABE) assay that detects S-palmitoylation of cysteine residues (Wan et al., 2007). After replacing thioester-linked-palmitate for biotin, formerly S-palmitoylated proteins are affinity-purified using Streptavidin beads and are detected by immunoblotting (Fig. 2A). To validate this assay, we analyzed extracts from embryos expressing either cytosolic GFP or palmitoylated YFP (palm-YFP) in tracheal cells. Indeed, we detected S-palmitoylation of palm-YFP, but not of GFP (Fig. 2B). We then tested the three known TCJ proteins and found that M6, but not Aka or Gli, was palmitoylated in embryos (Fig. 2C). M6 is predicted to be palmitoylated at a conserved cluster of three cysteine residues in the N-terminal cytoplasmic portion that is common to all M6 isoforms (Fig. 2D). The corresponding cysteine residues are palmitoylated in mouse GPM6a (Honda et al., 2017). We mutated the three cysteine residues to serine residues in UAS-M6–GFP and tested for palmitoylation of the mutant protein (referred to as *M6^3xCS^*) in transfected S2R+ cells. Whereas palmitoylation was readily detectable on wild-type M6–GFP, it was absent from M6^3xCS^–GFP (Fig. 2F), indicating that M6 is palmitoylated on at least one of the three cysteine residues and that this is the only site of S-palmitoylation in M6.

### Palmitoylation promotes accumulation of M6 at vertices

**Fig. 3.**
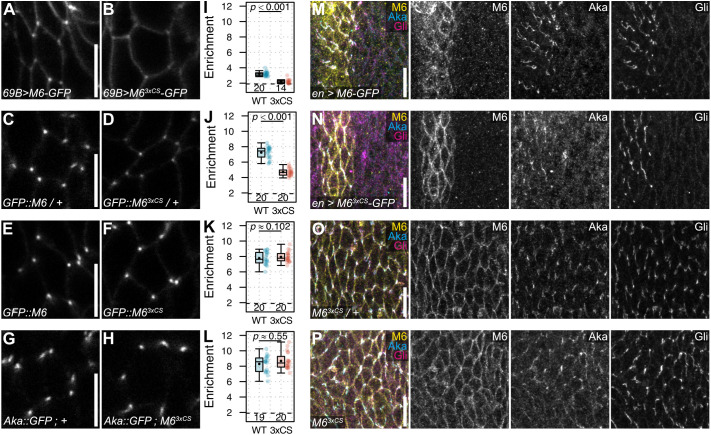
**Palmitoylation promotes but is not essential for vertex localization of M6.** (A–H) *En face* views of epidermis in living embryos (stage 15) overexpressing wild-type M6–GFP or palmitoylation-deficient M6^3xCS^–GFP under control of *69B*-Gal4 (A,B), or expressing endogenous wild-type GFP::M6 (C,E), GFP::M6^3xCS^ (D,F), or Aka::GFP (G, H). (A,B) When overexpressed in epidermis under control of *69B*-Gal4, M6–GFP (A) shows stronger enrichment at vertices compared to M6^3xCS^–GFP (B). (C,D) In heterozygous embryos carrying one wild-type copy of untagged M6, endogenous GFP::M6 (C) shows stronger enrichment at vertices compared to GFP::M6^3xCS^ (D). (E,F) In homozygous embryos lacking untagged M6, endogenous GFP::M6 and GFP::M6^3xCS^ show indistinguishable enrichment at vertices. (G,H) Vertex enrichment of Aka::GFP in control embryos (G) is indistinguishable from that in *M6^3xCS^* homozygous embryos (H). (I–L) Quantification of vertex enrichment of GFP signals in the indicated genotypes. Each datapoint represents the mean of 3 to 8 vertices in one embryo. Number of embryos (*n*) and *P*-values are indicated. Two-tailed unpaired Student's *t*-test (J,K) or two-tailed unpaired Wilcoxon rank-sum test (I,L). (M,N) *En face* view of lateral epidermis in M6-deficient (*M6^MB02608^/Df(3L)BSC419*) embryos (stage 15) expressing UAS-M6–GFP (M) or UAS- M6^3xCS^–GFP (N) in epidermal stripes under control of *en*-Gal4. Embryos were fixed and immunostained against Aka, M6 and Gli. Note that palmitoylation-deficient M6^3xCS^ rescues TCJ localization of Gli and partially rescues Aka localization in *en*-Gal4-expressing cells. (O,P) *En face* view of lateral epidermis in fixed embryos (stage 15) heterozygous (*M6^3xCS^/+*; O) or homozygous (*M6^3xCS^/M6^3xCS^*; P) for *M6^3xCS^*. Embryos were immunostained against Aka, M6 and Gli. Note that TCJ localization of Gli is normal and Aka localization is only mildly affected in *M6^3xCS^* homozygotes (P). Three independent repeats were carried out for each rescue experiment shown in M–P. Box plots show maximum and minimum observations within 1.5× interquartile range from the first and third quartile boundaries, upper and lower quartile, median (horizontal line) and mean (black dot). Scale bars: 5 µm (A,C,E,G); 10 µm (M–P).

Having established a palmitoylation-deficient M6 mutant, we asked whether palmitoylation is involved in vertex localization of M6. Therefore, we expressed either wild-type M6–GFP or *M6^3xCS^*–GFP in the embryonic epidermis (using *69B*-Gal4) and analyzed the subcellular distribution of the GFP-tagged proteins (Fig. 3A,B). *M6^3xCS^*–GFP was present at the plasma membrane at similar levels to those observed for wild-type M6–GFP (Fig. S2), indicating that palmitoylation is not required for intracellular trafficking or membrane targeting of M6, unlike other proteins, such as Wingless (Wg), which require palmitoylation for their secretion (Franch-Marro et al., 2008). However, *M6^3xCS^*–GFP showed significantly lower (2.2±0.3-fold) vertex enrichment compared to wild-type M6–GFP (3.2±0.3; mean±s.d., *P*<0.001; *n*=20 embryos; Fig. 3I), suggesting that palmitoylation promotes targeting of M6 to or stabilization at vertices.

To test whether M6 palmitoylation is required for TCJ formation, we expressed M6^3xCS^–GFP in epidermal stripes of *M6*-deficient embryos and analyzed the distribution of Aka and Gli (Fig. 3M,N). Surprisingly, Aka localization was partially rescued and Gli localization was fully rescued in M6^3xCS^–GFP-expressing cells (Fig. 3N), suggesting that M6 palmitoylation is dispensable for TCJ assembly, despite the low enrichment of M6^3xCS^–GFP at vertices. However, in this experiment, M6^3xCS^ protein was overexpressed, thus impeding conclusions about functional roles of M6 palmitoylation.

### Palmitoylated M6 outcompetes palmitoylation-deficient M6 from localizing to vertices

To analyze functions of M6 palmitoylation without overexpression, we mutated the cysteine cluster in the endogenous *M6* locus (with or without the GFP-trap insertion, referred to as *GFP::M6^3xCS^* or *M6^3xCS^*, respectively; Fig. 2E). Endogenous GFP::M6^3xCS^ protein showed significantly lower enrichment (4.7±0.5-fold; mean±s.d.) than wild-type GFP::M6 (7.2±0.8-fold; *P*<0.001; *n*=20 embryos) at epidermal cell vertices in heterozygous (*GFP::M6^3xCS^/+*) embryos (Fig. 3C,D,J), resembling the distribution of overexpressed M6^3xCS^–GFP (Fig. 3B). Surprisingly, however, in homozygous *GFP::M6^3xCS^* embryos, vertex enrichment of GFP::M6^3xCS^ was indistinguishable from that of wild-type GFP::M6 protein (Fig. 3E,F,K), suggesting that, in the heterozygous situation, wild-type M6 protein outcompetes GFP::M6^3xCS^ from occupying vertices. Despite this effect, TCJ localization of Aka and Gli appeared normal in *GFP::M6^3xCS^* heterozygous and homozygous embryos (Fig. 3O,P), and homozygous *GFP::M6^3xCS^* flies were viable and fertile. Thus, although palmitoylation is not essential for vertex localization of M6, it promotes transport to or maintenance of M6 at vertices.

### TCJ localization of M6 is delayed in the absence of palmitoylation

**Fig. 4.**
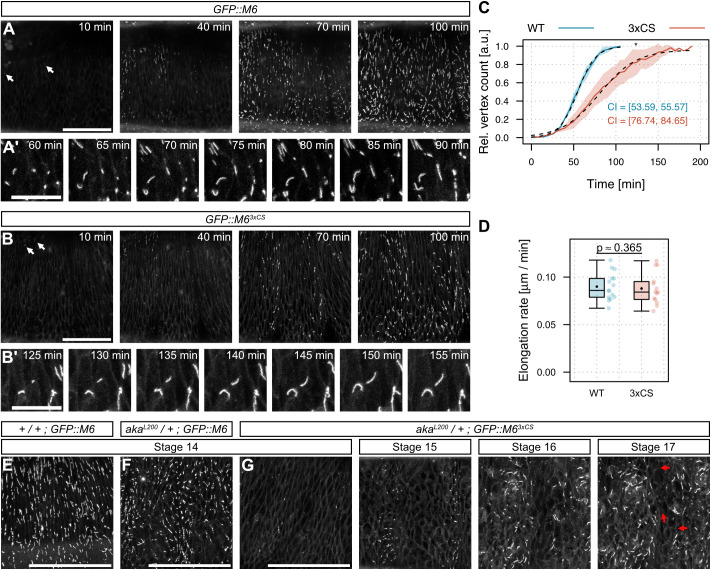
**Lack of M6 palmitoylation leads to delayed vertex accumulation of M6 but does not affect the rate of extension along vertices.** (A,B) *En face* view of dorso-lateral epidermis in living embryos (stage 13) homozygous for either *GFP::M6* (A) or *GFP::M6^3xCS^* (B). White arrows denote first appearance of GFP::M6 accumulation at vertices. Maximum intensity projections are shown. See also Movie 2. (A′,B′) Close-up showing extension of GFP::M6 (A′) or *GFP::M6^3xCS^* (B′) signals along vertices. Time is indicated. Rate of extension is quantified in D. (C) Quantification of vertex accumulation of GFP::M6 (blue) and GFP::M6^3xCS^ (red). The number of GFP-positive vertices was determined at each timepoint and normalized to the maximum number of GFP-positive vertices at the end of each movie. Note that GFP::M6 accumulated at most vertices throughout the epidermis within 100 min, whereas vertex accumulation of GFP::M6^3xCS^ was delayed. Dashed lines indicate fitted logistic model. 95% confidence intervals (CI; upper and lower limit) for time of half-maximal accumulation are indicated. A non-overlap indicates a statistical difference with *P*<0.05 (*). *n*=3 embryos per genotype. a.u., arbitrary units. (D) Quantification of extension rate of GFP::M6 (blue) and GFP::M6^3xCS^ (red) along vertices. Note that GFP::M6 and GFP::M6^3xCS^ extend with similar rates. *n*=15 vertices from 3 embryos per genotype. *P*-value is indicated (one-tailed *t*-test). (E–G) *En face* view of living embryos (stage 14). GFP::M6 occupies all vertices by stage 14 in wild-type (E) and *aka^L200^* heterozygotes (F), whereas GFP::M6^3xCS^ accumulates only at a small number of vertices at stage 14 in *aka^L200^/+* heterozygotes (G). Note that many vertices fail to accumulate (red arrows) GFP::M6^3xCS^ even in late (stage 17) embryos. Box plots show maximum and minimum observations within 1.5× interquartile range from the first and third quartile boundaries, upper and lower quartile, median (horizontal line) and mean (black dot). Scale bars: 50 µm (A, B, E–G); 2 µm (A′,B′).

To test the hypothesis that palmitoylation mediates localization of M6 at vertices, we analyzed the dynamics of GFP::M6 accumulation at epidermal cell vertices in time-lapse movies (Fig. 4A). At the onset of TCJ formation, GFP::M6 accumulates at the apical tip of each vertex in a single spot that subsequently extends basally with a speed of 0.09 µm/min (*n*=15 vertices in three embryos; Fig. 4A′,D; Movie 1; Wittek et al., 2020). This process starts at a small number of vertices during stage 13 and subsequently sweeps across the epidermis over the course of 95±10 min (*n*=3 embryos) until all vertices are occupied by GFP::M6 during stage 14 (Fig. 4A,C; Movie 2; Wittek et al., 2020). To quantify the kinetics of M6 vertex localization, we determined in each movie frame the fraction of vertices marked by accumulation of GFP::M6 (Fig. 4A). Whereas wild-type GFP::M6 occupied all vertices at 95±10 min (*n*=3 embryos) after the onset of TCJ formation, GFP::M6^3xCS^ reached complete vertex occupancy with a delay of 76% after 167±21 min (delay of 26 min in half-maximal occupancy time, *P*<0.05; Fig. 4B,C; Movie 2), indicating that efficient TCJ localization of M6 depends on palmitoylation.

### M6 palmitoylation promotes initial accumulation, but not extension of M6 clusters at vertices

Interestingly, although the rate of vertex occupancy by GFP::M6 was impaired by the lack of M6 palmitoylation, there was no detectable effect on the rate of extension along a vertex after GFP::M6 had accumulated at its apical tip (0.088 µm/min; *n*=15 vertices from three embryos; Fig. 4D). A similar delay in initial vertex accumulation, but not in subsequent extension along vertices, was observed for Aka::GFP in *M6^3xCS^* mutant embryos (Fig. S3). These findings suggest that efficient initial accumulation of Aka and M6 at vertices depends on M6 palmitoylation, whereas the subsequent extension of TCJ complexes during TCJ growth is independent of M6 palmitoylation. Consistent with this notion, the lack of M6 palmitoylation had only a slight, if any, effect on the mobility of M6 (GFP::M6^3xCS^) or of Aka (Aka::GFP) proteins at vertices, as measured by fluorescence recovery after photobleaching (FRAP) experiments (Fig. S4), whereas complete loss of M6 protein was previously shown to lead to substantially increased mobility of Aka and abolishes TCJ formation (Wittek et al., 2020).

### M6 palmitoylation becomes essential when dosage of Aka is reduced

Because vertex accumulation of M6 depends on Aka (Wittek et al., 2020), we wondered whether the delayed TCJ assembly in the absence of M6 palmitoylation can be further perturbed by reducing the dosage of Aka. Indeed, removing one copy of *aka* in the absence of M6 palmitoylation aggravated the delay in vertex occupancy of *GFP::M6^3xCS^*, with many vertices failing to accumulate M6 even in late-stage embryos (Fig. 4G), and led to synthetic embryonic lethality, indicating that M6 palmitoylation becomes essential when TCJ protein concentration is reduced. Taken together, these results highlight the requirement of M6 palmitoylation for robust TCJ assembly, and raised the question of whether Aka and M6 proteins interact with each other, possibly in a palmitoylation-dependent manner.

### M6 interacts with Aka and with itself

**Fig. 5.**
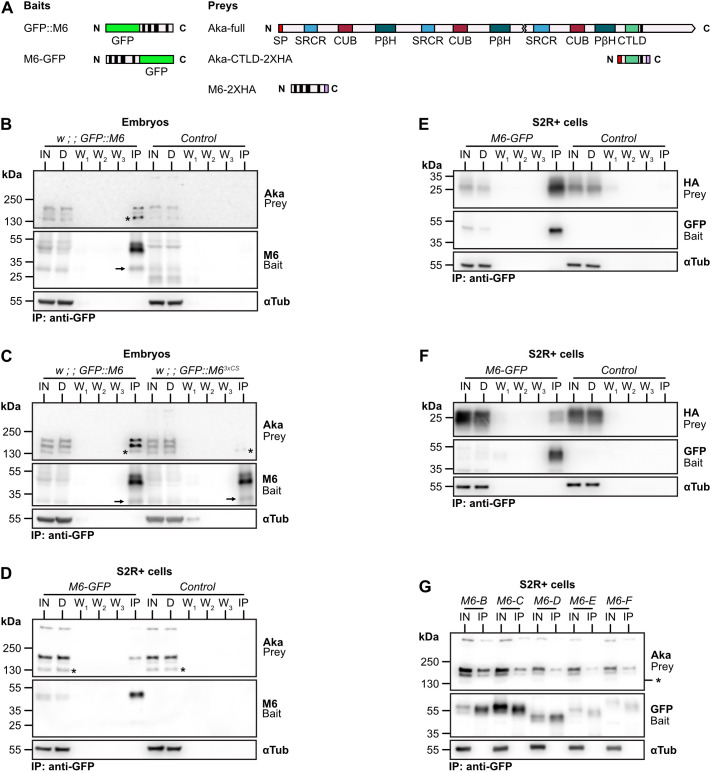
**M6 interacts with Aka and with itself.** (A) Schemes of proteins used as bait or prey in co-immunoprecipitation (co-IP) experiments. The extracellular portion of Aka comprises an N-terminal signal peptide (SP), scavenger receptor-cysteine-rich (SRCR) domains, complement C1r/C1s, UEGF, BMP1 (CUB) domains, right-handed parallel β-helices (PβH), and a C-type lectin-like domain (CTLD). Black bars indicate transmembrane domains. (B–G) IP experiments with lysates from embryos (B,C) or transfected S2R+ cells (D–G) expressing the indicated bait proteins. Immunoblots of input (IN; 5% of lysate), depleted input (labeled D, 5% of lysate), washes (W), and immunoprecipitation (IP) fractions were probed with antibodies indicated to the right. α-tubulin (αTub) was used as a loading control. Sizes (kDa) of a molecular mass marker are indicated to the left. A band at ∼130 kDa (marked by an asterisk) is non-specifically bound by the anti-Aka antiserum. At least three independent repeats were performed for each IP experiment shown. (B) IP with anti-GFP antibodies to immunoprecipitate GFP::M6. Note that GFP::M6 co-precipitates Aka and the non-GFP-tagged M6-B isoform (arrow) from lysates of embryos expressing GFP::M6 but not from negative control (*y w*) embryos. (C) IP of wild-type GFP::M6 (left) or palmitoylation-deficient GFP::M6^3xCS^ (right). Note that co-precipitation of Aka is strongly reduced with GFP::M6^3xCS^ compared to GFP::M6, whereas co-precipitation of M6-B (black arrow) is unchanged, indicating that the Aka–M6 interaction is palmitoylation dependent, whereas M6–M6 interactions are palmitoylation independent. (D) IP experiment with S2R+ cells co-transfected either with M6-GFP (bait) and Aka (prey; left) or with Aka alone (control; right). Note that M6–GFP specifically co-precipitates Aka. (E) IP experiment with S2R+ cells co-transfected either with M6-D–GFP (bait) and M6-D–2×HA (prey; left) or with M6-D–2×HA alone (control; right). Note that M6-D–GFP specifically co-precipitates M6-D–2XHA, indicating that M6 self-interacts. (F) A short fragment of Aka (scheme in A) comprising the extracellular C-type lectin-like domain (CTLD), the TM domain and a cytosolic 2×HA tag was co-transfected with UAS-M6–GFP into S2R+ cells. M6 specifically co-precipitates the short Aka fragment. (G) Each M6 isoform (isoforms B, C, D, E and F) was tagged with GFP (bait) and was individually co-transfected with Aka (prey) into S2R+ cells. Note that all M6 isoforms co-precipitate Aka.

To test for potential interactions between Aka and M6 proteins, we carried out co-immunoprecipitation (co-IP) experiments using GFP-tagged M6 as a bait protein (Fig. 5A). We found that GFP::M6 specifically co-precipitated Aka from embryo extracts (Fig. 5B), suggesting that Aka and M6 are part of a protein complex. This interaction was detected also in transfected S2R+ cells expressing UAS-Aka (prey) and UAS-M6–GFP (bait) but no other known junction components, suggesting that Aka and M6 interact directly (Fig. 5D,G). We further noticed that GFP::M6 specifically also co-precipitated the untagged M6-B isoform expressed in embryos (Fig. 5B, arrow). Likewise, M6–GFP co-precipitated 2×-hemagglutinin-tagged M6 (M6-2×HA) in extracts from transfected S2R+ cells (Fig. 5E), indicating that M6, like vertebrate GPM6a (Formoso et al., 2015), interacts with itself.

### M6 palmitoylation is required for M6–Aka interaction, but not for M6–M6 interaction

To test whether palmitoylation of M6 influences its binding to Aka or to itself, we performed co-IP experiments with extracts from *GFP::M6^3xCS^* embryos (Fig. 5C) or from *M6^3xCS^*–GFP-transfected S2R+ cells (Fig. 5D). Interestingly, the Aka–M6 interaction was strongly reduced or absent (a weakly co-precipitating band at 130 kDa is bound non-specifically by the anti-Aka antiserum), whereas the M6–M6 homotypic interaction was unaffected (Fig. 5C, arrow), suggesting that M6 interacts with Aka in a palmitoylation-dependent manner, but self-interacts in a palmitoylation-independent manner. Aiming to narrow down the M6-interacting site in Aka, we found that an Aka fragment comprising only the C-type-lectin domain (CLTD) and the TM domain (Aka-CTLD–2×HA) but lacking the remaining extracellular and cytosolic parts of Aka (Fig. 5A), still co-precipitated with M6–GFP (Fig. 5F). This suggests that Aka and M6 interact either via their TM domains or via the CTLD of Aka and implies that the palmitoylated cytosolic cysteine cluster in M6 is not the site of interaction with Aka. Instead, M6 palmitoylation appears to influence the interaction with Aka in an allosteric fashion.

## DISCUSSION

How specialized junctional complexes with distinct adhesive and occluding properties are built at three-cell contacts remains a fundamental open question in epithelial biology (Bosveld et al., 2018; Higashi and Chiba, 2020; Higashi and Miller, 2017). Three TCJ-specific proteins, Aka, Gli and M6, are known in *Drosophila*, but how they interact to assemble TCJs was not known. Here, we report insights into the early steps of TCJ assembly, which depends on interactions between the transmembrane proteins Aka and M6. We show first that all M6 isoforms share the elements required for vertex localization. Second, we demonstrate that M6 is palmitoylated on a conserved cysteine cluster and that this modification is required for efficient initial localization of M6 to vertices, but not for subsequent TCJ growth and maintenance, indicating that different mechanisms control the early and late phases, respectively, of TCJ formation. Third, we show that Aka interacts with M6 in a palmitoylation-dependent manner, whereas M6 interacts with itself independently of palmitoylation, possibly forming homotypic clusters like the mammalian M6 homolog GPM6a.

These findings provide a molecular basis for the interdependent localization of Aka and M6 at TCJs, although it is not yet clear which physical or chemical features of vertices mediate localization of proteins to these sites. Aka engages in homotypic trans-interactions that depend on its expression in three adjacent cells and on its large extracellular domain, but not on its cytoplasmic domain, for accumulating at vertices (Byri et al., 2015). This suggests that the specific geometry of vertices or the presence of three adjacent plasma membranes act to recruit or maintain Aka localization. M6 is required for vertex localization of Aka in a permissive fashion (Wittek et al., 2020). Our findings suggest possible mechanisms underlying this role. In one scenario, M6 might stabilize Aka in the plasma membrane by preventing its internalization. Supporting this idea is the finding that in the absence of M6 Aka was barely detectable at the plasma membrane but instead distributed throughout the cytoplasm (Wittek et al., 2020). Alternatively, association with M6 might concentrate Aka in a vertex-specific plasma membrane domain and thereby facilitate trans-interactions between Aka molecules on adjacent cells. This is reminiscent of the role of tetraspanins, which interact laterally among themselves and with other proteins to form large assemblies called tetraspanin-enriched microdomains (TEMs; Hemler, 2003) or a tetraspanin web (Lagaudrière-Gesbert et al., 1997) that controls the clustering and activity of TM proteins, such as EGFR or integrins (reviewed in van Deventer et al., 2017).

We identified S-palmitoylation as a post-translational modification, which, although not essential for vertex localization of M6 or for TCJ formation, significantly enhances the efficiency and robustness of this process. Supporting this notion, palmitoylation of M6 becomes essential for TCJ formation and viability under conditions of reduced TCJ protein concentration. How could palmitoylation promote targeting of M6 to vertices? In many TM proteins, including claudins (Van Itallie et al., 2005), tetraspanins (Yang et al., 2004) and mammalian GPM6a (Honda et al., 2017), palmitoylation of juxtamembrane cysteine residues mediates association with cholesterol-rich membrane domains (Blaskovic et al., 2013). Negative plasma membrane curvature at vertices might favor accumulation of cholesterol (Yesylevskyy et al., 2017) and could thereby attract palmitoylated proteins. Consistent with this idea, methyl-β-cyclodextrin-induced depletion of cholesterol from the plasma membrane impaired the vertex localization of palmitoylated angulin-1 in cultured mammalian cells (Oda et al., 2020), although direct evidence for elevated cholesterol levels at cell vertices is lacking thus far.

Lack of M6 palmitoylation reduces, although it does not completely abolish, its interaction with Aka, resulting in delayed accumulation of M6 and Aka at vertices. How could palmitoylation affect the interaction of M6 with Aka? Structural predictions using AlphaFold (Jumper et al., 2021) and PPM3 (Predicting Protein position in Membranes; Lomize et al., 2022) suggest that M6 protein adopts a tilted orientation in the membrane (Fig. S5). Palmitoylation of the juxtamembrane cysteine cluster could stabilize the tilt and help to resolve a potential hydrophobic mismatch resulting from the different lengths of the four TMDs, thereby promoting a confirmation of M6 that is favorable for interaction with Aka. Palmitoylation of LRP6 has been proposed to act in this way by resolving a hydrophobic mismatch that otherwise causes the protein to be retained in the ER (Abrami et al., 2008). Similarly, tetraspanins (e.g. CD151), require palmitoylation for interaction with other TM proteins (Zevian et al., 2011).

Intriguingly, eliminating palmitoylation of M6 revealed a distinct requirement of this modification for efficient initial accumulation of M6 at vertex tips, whereas the subsequent extension of these clusters along the apical–basal vertex axis apparently relies on a different mechanism. This might include homotypic M6–M6 interactions, which we found to be independent of M6 palmitoylation and which might aid in organizing an M6-enriched plasma membrane domain, reminiscent of TEMs (Hemler, 2003). Interestingly, in mammalian cells, palmitoylation of juxtamembrane cysteine residues has been proposed to play an analogous role in targeting angulin-1 to cholesterol-enriched membrane domains at vertices, where angulin-1 recruits tricellulin (Oda et al., 2020). However, angulin-1 vertex localization appears to be dispensable for maintaining tricellulin localization after its initial recruitment to TCJs (Oda et al., 2020), suggesting that tricellulin is maintained at vertices through association with other factors, for example, with claudin-based TJ strands (Ikenouchi et al., 2008). Analogously, in *Drosophila*, M6 palmitoylation is required early during TCJ formation for efficient targeting of M6 and Aka to vertices, whereas Aka is subsequently stabilized at TCJs through interaction with components of SJ limiting strands, including Gli (Esmangart de Bournonville and Le Borgne, 2020). Thus, although TCJs of vertebrates and invertebrates comprise different sets of proteins, our findings suggest that in both cases juxtamembrane palmitoylation contributes to targeting TM proteins to cell vertices during TCJ assembly.

## MATERIALS AND METHODS

### *Drosophila* husbandry

*Drosophila* was maintained on cornmeal-agar medium with added dry yeast. Embryos were collected on apple juice agar plates at 22°C or 25°C. The sex of embryos was not assessed.

### *Drosophila* strains and genetics

*Drosophila* stocks are described in FlyBase (Thurmond et al., 2019), unless noted otherwise: *aka^L200^* (Byri et al., 2015), *M6^CA06602^* (Buszczak et al., 2007), *M6^MB02608^* (Bellen et al., 2011), *aka::GFP*, *UAS-M6*, *UAS-M6-GFP* (Wittek et al., 2020), *UAS-Nrg^167^-GFP* (Byri et al., 2015), *UAS-GFP, UAS-palm-YFP*, *UAS-mCherry-nls, 69B-Gal4*, *btl*-Gal4, *en-Gal4, Df(3L)BSC419*, *CyO Dfd-GMR-nvYFP, TM6b Dfd-GMR-nvYFP* (Le et al., 2006), *TM2 Delta2-3*, *nos-Cas9*.

### Genome editing

A CRISPR-Cas9-based strategy was used to mutate the three clustered cysteine residues to serine residues in the *M6* locus of the *GFP::M6^CA06602^* line to yield *GFP::M6^3xCS^*. The sgRNA sequence 5′-GAATACGCGCCATGCAGGAT-3′ was cloned in the pCFD3 vector (Port et al., 2014). The resulting sgRNA plasmid (300 ng/µl) along with the ssDNA repair template (5′-ATTGCATTCAAAAGTTTATTGATATTATTTTTCCTAAAAATGTTTTTAGGAGAATcCTcgCAATCCTcCATGGCGCGTATTCCCTACGCCACCCTGATAGCCACTCTGATGTGTCTCCTG-3′; mutations underlined, 100 ng/µl, Eurofins Genomics) was injected into *nos-Cas9;; GFP::M6^CA06602^* embryos. Individual F_2_ males were screened for successful conversion by amplifying from genomic DNA of single adult flies a 795 bp fragment using a forward primer (5′-GAGAATcCTcgCAATCCTcCAT-3′) that is specific to the mutated sequence and a reverse primer (5′-GCTCCTGGAACTTTTCGTGG-3′). Positive candidates were analyzed by sequencing the target region. One line containing all four mutations was kept. In this line, the *w^+^*-marked GFP-trap P-element (P{PTT-GA}; Buszczak et al., 2007) was excised by P-element transposase to generate non-GFP-tagged *M6^3xCS^*.

### Molecular biology

Coding sequences for each annotated *M6* isoform (FlyBase) were synthesized (GenScript) with a 5′ EcoRI site and 3′ (GGS)_5_ linker followed by a NotI site. *M6* isoforms were fused either with the GFP coding sequence (flanked by 5′ NotI and 3′ XbaI sites) or with a 2×HA tag sequence (flanked by 5′ NotI and 3′ XbaI sites) and subcloned into EcoRI/XbaI digested pUASt-attB vector (Bischof et al., 2007), such that each M6 isoform was tagged C-terminally either with GFP or with 2XHA.

Point mutations to change cysteine residues to serine residues were inserted into the pUASt-attB-M6-GFP plasmid (Wittek et al., 2020) using site-directed mutagenesis. The plasmid was amplified using mutation-bearing oligonucleotides (forward primer: 5′-GAGAATcCTcCCAATCCTcCATGGCGC-3′; complementary reverse primer). Bacterial template DNA was digested with DpnI.

All UAS constructs were inserted into the attP2 genomic landing sites using PhiC31-mediated site-specific integration (Bischof et al., 2007).

### Cell culture

*Drosophila* S2R+ cells (*Drosophila* Genomics Resource Center) were cultured in Shields and Sang M3 medium (Biomol) supplemented with 1× penicillin-streptomycin (from 100× stock; Thermo Fisher Scientific) and 10% fetal calf serum (Thermo Fisher Scientific). 10^6^ cells were seeded per well in 24-well plates (Sarstedt) at 1 day before transfection. Cells were transfected with pCaSpeR4-Act5c-GAL4 plasmid (Byri et al., 2015, 600 ng) and pUASt-based expression plasmid (1000 ng) using FugeneHD reagent (Promega). Transfected cells were cultured at 25°C and processed within 1 to 3 days.

### Immunostaining

Embryos were dechorionated in sodium hypochlorite, washed, and fixed in 4% formaldehyde in PBS/heptane for 20 min at room temperature and then devitellinized by shaking in methanol/heptane. The following primary antibodies were used: guinea pig anti-M6 serum #1 (1:3000; Wittek et al., 2020), guinea pig anti-M6 serum #2 (1:3000; see below), rabbit anti-Aka (1:250; Byri et al., 2015) and mouse anti-Gli IF6.3 (1:500; Schulte et al., 2003). Goat secondary antibodies were conjugated to Alexa Fluor 488, Alexa Fluor 568 or Alexa Fluor 647 (1:500; Thermo Fisher Scientific). GFP signal was enhanced with FluoTag-X4 anti-GFP antibody (1:500; NanoTag).

### Antisera

The anti-M6 antiserum #2 was generated by immunizing guinea pigs (Eurogentec) with the peptides GKGNNRDRIRDPRE and RRNSYRSDHSLDRYT (corresponding to amino acids 57–71 and 102–116 in M6 isoform F) conjugated to keyhole limpet hemocyanin. Final serum was used at a dilution of 1:3000 for immunostainings.

### Acyl-biotin-exchange assay

The acyl-biotin-exchange (ABE) assay was adapted from (Wan et al., 2007). Approximately 200 embryos [16–20 hours after egg laying (hAEL)] were collected, dechorionated and homogenized using a 7.5 ml Dounce homogenizer in 1.2 ml lysis buffer containing 1.7% Triton X-100, 10 mM N-ethylmaleimide (NEM) and 1× protease inhibitor (Thermo Fisher Scientific). GFP-tagged M6 was purified using 20 µl GFP-Trap magnetic agarose (Chromotek). For other proteins, lysates were further processed without an intermediate purification step. Further steps including hydroxylamine (HA) treatment and biotinylation were carried out as described previously (Wan et al., 2007). Biotinylated proteins were purified using 10 µl streptavidin-coupled magnetic beads (Thermo Fisher Scientific) and eluted in 40 µl 1× Laemmli buffer at 70°C for 10 min. Samples were applied to SDS-PAGE followed by western blotting and staining for detection of palmitoylated proteins.

### Co-immunoprecipitation

Co-immunoprecipitation experiments were performed with extracts from embryos or from transfected S2R+ cells. Approximately 500 embryos (14 to 18 hAEL) were dechorionated and homogenized using a 7.5 ml Dounce homogenizer in 600 µl lysis buffer (1% Brij-97, 0.1% Tween-20, 10 mM Tris-HCl, 150 mM NaCl, 1 mM MgCl_2_, 1 mM CaCl_2_, 1× protease inhibitor; pH 7.4) on ice. S2R+ cells were lysed in 200 µl lysis buffer and otherwise treated like embryo extracts. Lysates were incubated at 4°C with end-over-end rotation for 30 min followed by centrifugation (2000 ***g***, 5 min, 4°C). Supernatants were incubated for 30 min with 20 µl GFP-Trap magnetic beads (Chromotek gtd-20) at 4°C with end-over-end rotation. Beads were washed three times with 200 µl lysis buffer. During the last washing step, beads were transferred to a new vial and NaCl concentration was raised to 300 mM. Proteins were eluted in 60 µl 1× Laemmli buffer by incubating for 10 min at 70°C.

### Immunoblotting

Samples were mixed 3:1 with 4× Laemmli buffer and incubated at 70°C for 10 min. 14 µl were loaded on 4%–20% Mini-PROTEAN TGX gels (Bio-Rad) and blotted on 0.45 µm PVDF membranes (Amersham). The following primary antibodies were used: mouse anti-GFP JL-8 (1:1000; TaKaRa), mouse anti-Tubulin AA4.3-c (1:5000; DSHB), rabbit anti-Aka (1:1000; Byri et al., 2015) and guinea pig anti-M6 serum #1 (1:2500; Wittek et al., 2020). Primary antibodies were detected with HRP-conjugated secondary antibodies (1:10000; Thermo Fisher) and ECL Prime kit (Amersham). Raw images were adjusted for brightness and contrast using ImageJ (Schindelin et al., 2012). Uncropped images of immunoblots are shown in the Fig. S6.

### Microscopy

Imaging was performed on a Leica SP8 confocal microscope with a 40×/1.3 NA oil immersion objective. For live imaging, dechorionated embryos were staged according to gut morphology, mounted on glue-coated coverslips (0.17 mm, grade #1.5), and covered with Voltalef 10S oil (VWR).

### Image analysis

Images were processed using either FIJI software (Schindelin et al., 2012) or Python 3.9 with the Scikit-Image package v0.18.2 (van der Walt et al., 2014). Image panels in figures were assembled using OMERO.figure v4.4.1 (Allan et al., 2012).

### Quantification of vertex enrichment

Enrichment of fluorescently tagged proteins at cell vertices was determined as follows. Embryos (stage 15) were aligned laterally and mounted as described above. *Z*-stacks (15 slices with 768×768px each) through epidermal cells were acquired at 100 Hz. In each stack, several cells were analyzed using a semiautomatic pipeline. Vertices were marked manually in the slice with highest vertex intensity and segmented by selecting a circular area of 5 px diameter around the marked point. Using the marked vertices as starting points the bicellular membrane was then segmented automatically using a self-written Python script (available upon request). For each cell, the mean intensity at vertices (*I*_*TCJ*_), the bicellular membrane (*I*_*BCJ*_), and in the cytoplasm (*I*_*BG*_) was measured (see Fig. 1C). Enrichment was calculated as:

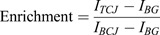


Per embryo, three to eight cells were selected and the mean for each embryo was calculated. Only vertices bordered by three cells but not four or more cells were considered. Sample size (*n*) indicated in the figures states the number of analyzed embryos.

### Quantification of M6–GFP signal intensities

To compare expression levels (GFP signal intensities) of UAS-M6–GFP constructs, image stacks of the lateral epidermis in stage 15 embryos were acquired. For each slice of the stack, a mask for the plasma membrane was created by applying first a median filter (1 px radius) and second a Gaussian filter (s.d.=1 px) before local thresholding was done using Niblacks algorithm with a windows size of 51 pixels. The binary image was further processed by applying binary erosion (1 px radius). The final stack of binary mask images was used to measure the mean intensity of the corresponding region in the original image. The relationship between signal intensity and enrichment factor was analyzed using a linear regression modelling the enrichment factor as a function of signal intensities. The dataset for the palmitoylation-deficient M6^3xCS^–GFP was not included in this model.

### Quantification of TCJ initiation and junction growth

Staged embryos were mounted dorso-laterally. The thorax region of embryos was imaged using a 40×/1.3 NA oil immersion objective and 2.5× zoom, 200 lines/s speed, 1528×1528 px resolution, 18 slices with a step-size of 0.5 µm, at 5 min intervals. Movies were processed frame by frame by applying a Gaussian filter (s.d. 1.5) followed by a white top-hat transform with a circular kernel of 9 px diameter. Movies were afterwards binarized using Otsu's threshold based on the stack at the last timepoint. Objects smaller than 10 px were filtered out. Afterwards, we counted the number of objects at each timepoint and normalized the count to the highest number of objects found at any timepoint yielding the relative vertex count. All movies were aligned temporally such that the first timepoint with a relative vertex count of at least 0.05 was set to 30 min. This data was used to fit a logistic regression model using the *nls* and *SSlogis* function of the R statistical software package (v4.1; https://www.r-project.org/). A 95% confidence interval was calculated for the *xmid* parameter, which represents the timepoint with a relative vertex count of 0.5. While an overlap of the intervals does not allow statements about statistical significance, a non-overlap indicates statistically significant differences in accumulation time to a test-niveau of α=0.05. Extension of GFP signals along the apico-basal vertex axis was analyzed at manually selected vertices that were oriented mostly parallel to the *xy*-plane and grew throughout the entire observation phase. TCJ length was measured in 3D shortly after initial accumulation and 30 min later by manually tracing GFP::M6 signal through *z*-stacks. TCJ growth rate was calculated by dividing the measured length increase by the observation time.

### Fluorescence recovery after photobleaching

Stage 15 embryos were selected according to gut morphology and were mounted dorsally. Movies were acquired using a 40×/1.3 NA oil immersion objective using the FRAP wizard of the LAS X software (Leica) with a speed of 400 lines/s and 2× line accumulation. For each embryo, three pre-bleach stacks (each with four *z*-slices) were acquired followed by three successive bleach rounds at 100% laser intensity, followed by 41 post-bleach stacks. The interval between pre- and post-bleach stacks was 30 s. *Z*-shift was corrected manually during acquisition. Given that the FRAP data of vertices is not suitable for classical FRAP analysis, we used a simplified approach to model the recovery. For each timepoint *t* we generated average intensity projections of slices 2–4. The vertex of interest was selected manually in each frame. We measured the intensities using circular regions of interest with a radius of 5 pixels. The FRAP values were calculated as follows. Let be *I*_*t*_, *t*∈{−3,−2, …, 40}, the raw intensities at timepoint *t* (bleached between *t*=− 1 and *t*=0). Let be *I*_0.95,*t*_ the 95% intensity quantile of the frame (to account for bleaching during acquisition) and *b*_*t*_ the background intensity at timepoint t. Then the corrected intensities *C*_*t*_ were calculated as:

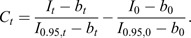
The normalized intensities *N*_*t*_ were calculated as:

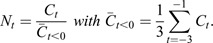
The graphs show the mean values of *N*_*t*_±*SD*_*t*_. The recovery function *f*_*A*,*τ*_ is defined as:

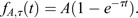
Here, *A* represents the mobile fraction and τ defines the fluorescence recovery rate. *A* and τ were fitted numerically with a non-linear least square approach using the *nls* function of the R statistical software package (v4.1). The mobile fraction was directly inferred from *A*. The half-recovery time was calculated as 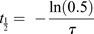.

### Statistics

For phenotypic analyses, sample size (*n*) was not predetermined using statistical methods, but was assessed by considering the variability of a given phenotype, determined by the standard deviation. Experiments were considered independent if the specimens analyzed were derived from different parental crosses. During experiments investigators were aware of allocation groups. Sample groups were checked for normality using the Shapiro–Wilk test and for equal variances using an *F* test. In case of normality and equal variances a pairwise two- or one-tailed *t*-test was applied. In case of unequal variances, a *t*-test using pooled standard deviations was used (Welch test). If data was not normally distributed, a Wilcoxon rank-sum test was applied. If samples were used for several tests, a *P*-value correction according to Holm (Holm, 1979) was applied to correct for multiple testing.

## Supplementary Material



10.1242/joces.261916_sup1 Supplementary information

## References

[JCS261916C1] Abrami, L., Kunz, B., Iacovache, I. and van der Goot, F. G. (2008). Palmitoylation and ubiquitination regulate exit of the Wnt signaling protein LRP6 from the endoplasmic reticulum. *Proc. Natl. Acad. Sci. USA* 105, 5384-5389. 10.1073/pnas.0710389105 18378904 PMC2291096

[JCS261916C2] Aimon, S., Callan-Jones, A., Berthaud, A., Pinot, M., Toombes, G. E. S. and Bassereau, P. (2014). Membrane shape modulates transmembrane protein distribution. *Dev. Cell* 28, 212-218. 10.1016/j.devcel.2013.12.012 24480645 PMC3954780

[JCS261916C3] Allan, C., Burel, J.-M., Moore, J., Blackburn, C., Linkert, M., Loynton, S., MacDonald, D., Moore, W. J., Neves, C., Patterson, A. et al. (2012). OMERO: flexible, model-driven data management for experimental biology. *Nat. Methods* 9, 245-253. 10.1038/nmeth.1896 22373911 PMC3437820

[JCS261916C4] Bellen, H. J., Levis, R. W., He, Y., Carlson, J. W., Evans-Holm, M., Bae, E., Kim, J., Metaxakis, A., Savakis, C., Schulze, K. L. et al. (2011). The Drosophila gene disruption project: progress using transposons with distinctive site specificities. *Genetics* 188, 731-743. 10.1534/genetics.111.126995 21515576 PMC3176542

[JCS261916C5] Bischof, J., Maeda, R. K., Hediger, M., Karch, F. and Basler, K. (2007). An optimized transgenesis system for Drosophila using germ-line-specific C31 integrases. *Proc. Natl. Acad. Sci. USA* 104, 3312-3317. 10.1073/pnas.0611511104 17360644 PMC1805588

[JCS261916C6] Blaskovic, S., Blanc, M. and Goot, F. G. and van der , (2013). What does S-palmitoylation do to membrane proteins? *FEBS J..* 280, 2766-2774. 10.1111/febs.12263 23551889

[JCS261916C7] Bosveld, F., Wang, Z. and Bellaïche, Y. (2018). Tricellular junctions: a hot corner of epithelial biology. *Curr. Opin. Cell Biol.* 54, 80-88. 10.1016/j.ceb.2018.05.002 29843079

[JCS261916C8] Burns, A. R., Walker, D. C., Brown, E. S., Thurmon, L. T., Bowden, R. A., Keese, C. R., Simon, S. I., Entman, M. L. and Smith, C. W. (1997). Neutrophil transendothelial migration is independent of tight junctions and occurs preferentially at tricellular corners. *J. Immunol.* 159, 2893-2903. 10.4049/jimmunol.159.6.2893 9300713

[JCS261916C9] Buszczak, M., Paterno, S., Lighthouse, D., Bachman, J., Planck, J., Owen, S., Skora, A. D., Nystul, T. G., Ohlstein, B., Allen, A. et al. (2007). The Carnegie Protein Trap Library: A Versatile Tool for Drosophila Developmental Studies. *Genetics* 175, 1505-1531. 10.1534/genetics.106.065961 17194782 PMC1840051

[JCS261916C10] Byri, S., Misra, T., Syed, Z. A., Bätz, T., Shah, J., Boril, L., Glashauser, J., Aegerter-Wilmsen, T., Matzat, T., Moussian, B. et al. (2015). The triple-repeat protein Anakonda controls epithelial tricellular junction formation in Drosophila. *Dev. Cell* 33, 535-548. 10.1016/j.devcel.2015.03.023 25982676

[JCS261916C11] Chamberlain, L. H. and Shipston, M. J. (2015). The physiology of protein S-acylation. *Physiol. Rev.* 95, 341-376. 10.1152/physrev.00032.2014 25834228 PMC4551212

[JCS261916C12] Esmangart de Bournonville, T. and Le Borgne, R. (2020). Interplay between Anakonda, Gliotactin, and M6 for tricellular junction assembly and anchoring of septate junctions in Drosophila epithelium. *Curr. Biol.* 30, 4245-4253.e4. 10.1016/j.cub.2020.07.090 32857971

[JCS261916C13] Formoso, K., García, M. D., Frasch, A. C. and Scorticati, C. (2015). Filopodia formation driven by membrane glycoprotein M6a depends on the interaction of its transmembrane domains. *J. Neurochem.* 134, 499-512. 10.1111/jnc.13153 25940868

[JCS261916C14] Franch-Marro, X., Wendler, F., Griffith, J., Maurice, M. M. and Vincent, J.-P. (2008). In vivo role of lipid adducts on Wingless. *J. Cell Sci.* 121, 1587-1592. 10.1242/jcs.015958 18430784 PMC7611555

[JCS261916C15] Fristrom, D. K. (1982). Septate junctions in imaginal disks of Drosophila: a model for the redistribution of septa during cell rearrangement. *J. Cell Biol.* 94, 77-87. 10.1083/jcb.94.1.77 7119018 PMC2112176

[JCS261916C16] Graf, F., Noirot-Timothée, C. and Noirot, C. (1982). The specialization of septate junctions in regions of tricellular junctions: I. Smooth septate junctions (=Continuous junctions). *J. Ultrastruct. Res.* 78, 136-151. 10.1016/S0022-5320(82)80019-1 7086932

[JCS261916C17] Hemler, M. E. (2003). Tetraspanin proteins mediate cellular penetration, invasion, and fusion events and define a novel type of membrane microdomain. *Annu. Rev. Cell Dev. Biol.* 19, 397-422. 10.1146/annurev.cellbio.19.111301.153609 14570575

[JCS261916C18] Higashi, T. and Chiba, H. (2020). Molecular organization, regulation and function of tricellular junctions. *Biochim. Biophys. Acta BBA Biomembr.* 1862, 183143. 10.1016/j.bbamem.2019.183143 31812626

[JCS261916C19] Higashi, T. and Miller, A. L. (2017). Tricellular junctions: how to build junctions at the TRICkiest points of epithelial cells. *Mol. Biol. Cell* 28, 2023-2034. 10.1091/mbc.e16-10-0697 28705832 PMC5509417

[JCS261916C20] Higashi, T., Tokuda, S., Kitajiri, S., Masuda, S., Nakamura, H., Oda, Y. and Furuse, M. (2013). Analysis of the ‘angulin’ proteins LSR, ILDR1 and ILDR2 – Tricellulin recruitment, epithelial barrier function and implication in deafness pathogenesis. *J. Cell Sci.* 126, 966-977. 10.1242/jcs.138271 23239027

[JCS261916C50] Hildebrandt, A., Pflanz, R., Behr, M., Tarp, T., Riedel, D. and Schuh, R. (2015). Bark beetle controls epithelial morphogenesis by septate junction maturation in Drosophila. *Dev. Biol.* 400, 237-247. 10.1016/j.ydbio.2015.02.008 25704509

[JCS261916C21] Holm, S. (1979). A simple sequentially rejective multiple test procedure. *Scand. J. Stat.* 6, 65-70.

[JCS261916C22] Honda, A., Ito, Y., Takahashi-Niki, K., Matsushita, N., Nozumi, M., Tabata, H., Takeuchi, K. and Igarashi, M. (2017). Extracellular Signals Induce Glycoprotein M6a Clustering of Lipid Rafts and Associated Signaling Molecules. *J. Neurosci.* 37, 4046-4064. 10.1523/JNEUROSCI.3319-16.2017 28275160 PMC6596588

[JCS261916C23] Ikenouchi, J., Furuse, M., Furuse, K., Sasaki, H., Tsukita, S. and Tsukita, S. (2005). Tricellulin constitutes a novel barrier at tricellular contacts of epithelial cells. *J. Cell Biol.* 171, 939-945. 10.1083/jcb.200510043 16365161 PMC2171318

[JCS261916C24] Ikenouchi, J., Sasaki, H., Tsukita, S., Furuse, M. and Tsukita, S. (2008). Loss of occludin affects tricellular localization of Tricellulin. *Mol. Biol. Cell* 19, 4687-4693. 10.1091/mbc.e08-05-0530 18768749 PMC2575184

[JCS261916C25] Jumper, J., Evans, R., Pritzel, A., Green, T., Figurnov, M., Ronneberger, O., Tunyasuvunakool, K., Bates, R., Žídek, A., Potapenko, A. et al. (2021). Highly accurate protein structure prediction with AlphaFold. *Nature* 596, 583-589. 10.1038/s41586-021-03819-2 34265844 PMC8371605

[JCS261916C26] Lagaudrière-Gesbert, C., Naour, F. L., Lebel-Binay, S., Billard, M., Lemichez, E., Boquet, P., Boucheix, C., Conjeaud, H. and Rubinstein, E. (1997). Functional analysis of four Tetraspans, CD9, CD53, CD81, and CD82, suggests a common role in costimulation, cell adhesion, and migration: only CD9 upregulates HB-EGF activity. *Cell. Immunol.* 182, 105-112. 10.1006/cimm.1997.1223 9514697

[JCS261916C27] Le, T., Liang, Z., Patel, H., Yu, M. H., Sivasubramaniam, G., Slovitt, M., Tanentzapf, G., Mohanty, N., Paul, S. M., Wu, V. M. et al. (2006). A new family of drosophila balancer chromosomes with a w− dfd-GMR yellow fluorescent protein marker. *Genetics* 174, 2255-2257. 10.1534/genetics.106.063461 17057238 PMC1698648

[JCS261916C28] Lomize, A. L., Todd, S. C. and Pogozheva, I. D. (2022). Spatial arrangement of proteins in planar and curved membranes by PPM 3.0. *Protein Sci.* 31, 209-220. 10.1002/pro.4219 34716622 PMC8740824

[JCS261916C29] Masuda, S., Oda, Y., Sasaki, H., Ikenouchi, J., Higashi, T., Akashi, M., Nishi, E. and Furuse, M. (2011). LSR defines cell corners for tricellular tight junction formation in epithelial cells. *J. Cell Sci.* 124, 548-555. 10.1242/jcs.072058 21245199

[JCS261916C30] Noirot-Timothée, C., Graf, F. and Noirot, C. (1982). The specialization of septate junctions in regions of tricellular junctions: II. Pleated septate junctions. *J. Ultrastruct. Res.* 78, 152-165. 10.1016/S0022-5320(82)80020-8 7086933

[JCS261916C31] Oda, Y., Sugawara, T., Fukata, Y., Izumi, Y., Otani, T., Higashi, T., Fukata, M. and Furuse, M. (2020). The extracellular domain of angulin-1 and palmitoylation of its cytoplasmic region are required for angulin-1 assembly at tricellular contacts. *J. Biol. Chem.* 295, 4289-4302. 10.1074/jbc.RA119.010491 32079676 PMC7105312

[JCS261916C32] Papatheodorou, P., Carette, J. E., Bell, G. W., Schwan, C., Guttenberg, G., Brummelkamp, T. R. and Aktories, K. (2011). Lipolysis-stimulated lipoprotein receptor (LSR) is the host receptor for the binary toxin Clostridium difficile transferase (CDT). *Proc. Natl. Acad. Sci. USA* 108, 16422-16427. 10.1073/pnas.1109772108 21930894 PMC3182710

[JCS261916C33] Port, F., Chen, H.-M., Lee, T. and Bullock, S. L. (2014). Optimized CRISPR/Cas tools for efficient germline and somatic genome engineering in Drosophila. *Proc. Natl. Acad. Sci.* 111, E2967-E2976. 10.1073/pnas.1405500111 25002478 PMC4115528

[JCS261916C34] Riazuddin, S., Ahmed, Z. M., Fanning, A. S., Lagziel, A., Kitajiri, S., Ramzan, K., Khan, S. N., Chattaraj, P., Friedman, P. L., Anderson, J. M. et al. (2006). Tricellulin Is a Tight-Junction Protein Necessary for Hearing. *Am. J. Hum. Genet.* 79, 1040-1051. 10.1086/510022 17186462 PMC1698716

[JCS261916C35] Schindelin, J., Arganda-Carreras, I., Frise, E., Kaynig, V., Longair, M., Pietzsch, T., Preibisch, S., Rueden, C., Saalfeld, S., Schmid, B. et al. (2012). Fiji: an open-source platform for biological-image analysis. *Nat. Methods* 9, 676-682. 10.1038/nmeth.2019 22743772 PMC3855844

[JCS261916C36] Schulte, J., Tepass, U. and Auld, V. J. (2003). Gliotactin, a novel marker of tricellular junctions, is necessary for septate junction development in *Drosophila*. *J. Cell Biol.* 161, 991-1000. 10.1083/jcb.200303192 12782681 PMC2172969

[JCS261916C37] Staehelin, L. A. (1973). Further observations on the fine structure of freeze-cleaved tight junctions. *J. Cell Sci.* 13, 763-786. 10.1242/jcs.13.3.763 4203962

[JCS261916C38] Sumagin, R. and Sarelius, I. H. (2010). Intercellular adhesion molecule-1 enrichment near Tricellular endothelial junctions is preferentially associated with leukocyte transmigration and signals for reorganization of these junctions to accommodate leukocyte passage. *J. Immunol.* 184, 5242-5252. 10.4049/jimmunol.0903319 20363969 PMC2946363

[JCS261916C39] Sumitomo, T., Nakata, M., Higashino, M., Yamaguchi, M. and Kawabata, S. (2016). Group A Streptococcus exploits human plasminogen for bacterial translocation across epithelial barrier via tricellular tight junctions. *Sci. Rep.* 6, 20069. 10.1038/srep20069 PMC4731814 26822058

[JCS261916C40] Thurmond, J., Goodman, J. L., Strelets, V. B., Attrill, H., Gramates, L. S., Marygold, S. J., Matthews, B. B., Millburn, G. et al. (2019). FlyBase 2.0: the next generation. *Nucleic Acids Res.* 47, D759-D765. 10.1093/nar/gky1003 30364959 PMC6323960

[JCS261916C41] van der Walt, S., Schönberger, J. L., Nunez-Iglesias, J., Boulogne, F., Warner, J. D., Yager, N., Gouillart, E. and Yu, T. (2014). scikit-image: image processing in Python. *PeerJ* 2, e453. 10.7717/peerj.453 25024921 PMC4081273

[JCS261916C42] van Deventer, S. J., Dunlock, V.-M. E. and van Spriel, A. B. (2017). Molecular interactions shaping the tetraspanin web. *Biochem. Soc. Trans.* 45, 741-750. 10.1042/BST20160284 28620035

[JCS261916C43] Van Itallie, C. M., Gambling, T. M., Carson, J. L. and Anderson, J. M. (2005). Palmitoylation of claudins is required for efficient tight-junction localization. *J. Cell Sci.* 118, 1427-1436. 10.1242/jcs.01735 15769849

[JCS261916C44] Wan, J., Roth, A. F., Bailey, A. O. and Davis, N. G. (2007). Palmitoylated proteins: purification and identification. *Nat. Protoc.* 2, 1573-1584. 10.1038/nprot.2007.225 17585299

[JCS261916C45] Wittek, A., Hollmann, M., Schleutker, R. and Luschnig, S. (2020). The transmembrane proteins M6 and Anakonda cooperate to initiate Tricellular junction assembly in epithelia of Drosophila. *Curr. Biol.* 30, 4254-4262.e5. 10.1016/j.cub.2020.08.003 32857972

[JCS261916C46] Yang, X., Kovalenko, O. V., Tang, W., Claas, C., Stipp, C. S. and Hemler, M. E. (2004). Palmitoylation supports assembly and function of integrin–tetraspanin complexes. *J. Cell Biol.* 167, 1231-1240. 10.1083/jcb.200404100 15611341 PMC2172609

[JCS261916C47] Yesylevskyy, S. O., Rivel, T. and Ramseyer, C. (2017). The influence of curvature on the properties of the plasma membrane. Insights from atomistic molecular dynamics simulations. *Sci. Rep.* 7, 16078. 10.1038/s41598-017-16450-x 29167583 PMC5700167

[JCS261916C48] Zappia, M. P., Brocco, M. A., Billi, S. C., Frasch, A. C. and Ceriani, M. F. (2011). M6 membrane protein plays an essential role in Drosophila oogenesis. *PLoS ONE* 6, e19715. 10.1371/journal.pone.0019715 21603606 PMC3095610

[JCS261916C49] Zevian, S., Winterwood, N. E. and Stipp, C. S. (2011). Structure-function analysis of tetraspanin CD151 reveals distinct requirements for tumor cell behaviors mediated by α3β1 versus α6β4 Integrin *. *J. Biol. Chem.* 286, 7496-7506. 10.1074/jbc.M110.173583 21193415 PMC3045005

