# Supplementary material for: Palmitoylation of proteolipid protein M6 promotes tricellular junction assembly in epithelia of Drosophila
Source: J Cell Sci. 2024 Mar 20;137(6):jcs261916. doi: 10.1242/jcs.261916 (PMC11698045; doi:10.1242/jcs.261916)
Supplement: Supplementary information [file joces-137-261916-s1.pdf]

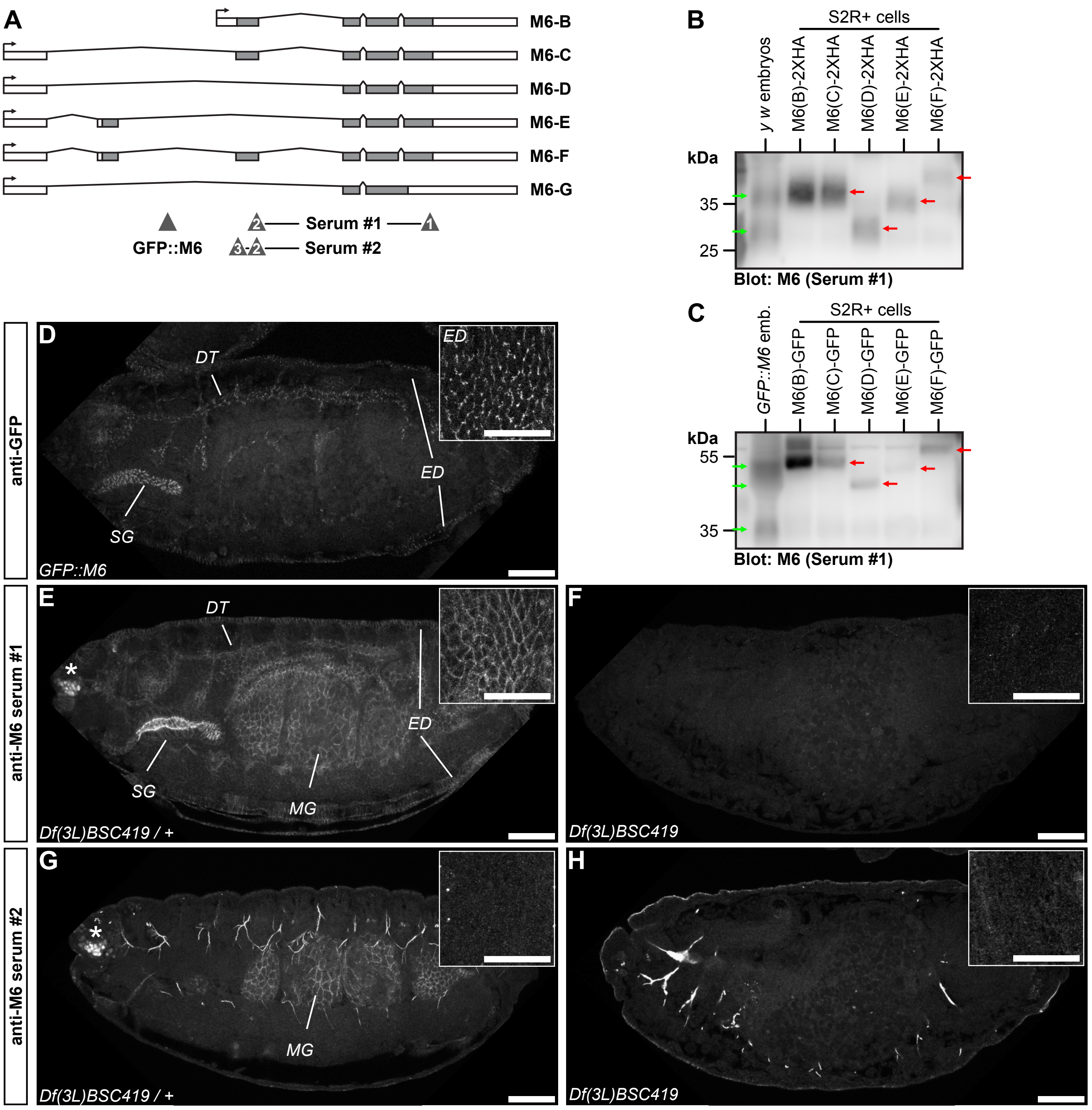

Fig. S1 (related to Fig. 1): Expression of M6 isoforms in embryos.

(A) Scheme of *M6* transcript isoforms. Insertion site of the GFP::M6 (M6^{CA06602}) protein trap transposon and positions of peptides used to generate anti-M6 serum #1 and #2 are indicated by triangles. The GFP::M6 protein trap tags M6 isoforms transcribed from the upstream promoter (all but isoform B). Predicted isoform G contains a retained intron and downstream premature stop codon, resulting in a predicted truncated protein, and was not further considered.

(B) Immunoblot of lysates from embryos (*y w*; 0-24 h after egg lay) or from S2R+ cells transfected with single HA-tagged M6 isoforms indicated on top. Two bands (green arrows) in the embryo lysate correspond to isoforms B or C (36 kDa) and D (28 kDa), respectively (red arrows).

(C) Immunoblot of lysates from embryos (0-24 h after egg lay) expressing endogenous GFP::M6 or from S2R+ cells transfected with the indicated GFP-tagged M6 isoform. Three bands (green arrows)

Journal of Cell Science • Supplementary information

correspond to untagged isoform B (lower band; compare panel A), tagged isoform D (middle band), and tagged isoform C (upper band).

(D) Embryo (stage 16) expressing endogenous GFP::M6 ($M6^{CA06602}$) immunostained against GFP. GFP::M6 tags M6 isoforms C, D, E, F, and G and is detected in ectodermal tissues, including epidermis (ED), salivary glands (SG) and tracheal dorsal trunk (DT), but not in endodermal midgut (MG). Inset shows *en face* view of epidermis of the same embryo. Note that anti-GFP staining detects GFP::M6 at cell vertices, as observed in living embryos.

(E, F) Embryos (stage 16) either heterozygous (E) or homozygous (F) for *Df(3L)BSC419* are shown. Immunostaining with anti-M6 serum #1 detects M6 expression in the ectoderm (epidermis, salivary gland, trachea), resembling distribution of the GFP::M6 protein trap (D), and additionally in the endoderm (midgut). Absence of signal in *Df(3L)BSC419* homozygous embryo (F) indicates specificity of the antiserum. Asterisk in (E) indicates YFP signal from the *Dfd-GMR-YFP* balancer chromosome used to genotype embryos.

(G, H) Embryos (stage 16) either heterozygous (G) or homozygous (H) for *Df(3L)BSC419* are shown. Immunostaining with anti-M6 serum #2 detects M6 expression in endoderm (midgut), but not in ectodermal tissues. Endodermal expression corresponds to M6-B, which is the only isoform not tagged by the GFP::M6 ($M6^{CA06602}$) protein trap. Signal in tracheal lumen in *Df(3L)BSC419* heterozygous (G) and homozygous (H) embryos is non-specific. Asterisk in (G) indicates YFP signal from the *Dfd-GMR-YFP* balancer chromosome used to genotype embryos. Note that compared to anti-GFP staining in GFP::M6 embryos (D), anti-M6 antisera #1 (E) and #2 (G) detect only little enrichment of M6 at TCJs, possibly because the respective epitopes might be inaccessible when M6 is incorporated into TCJs.

Scale bars: (D-H) overviews, 50 µm; insets, 25 µm.

Journal of Cell Science • Supplementary information

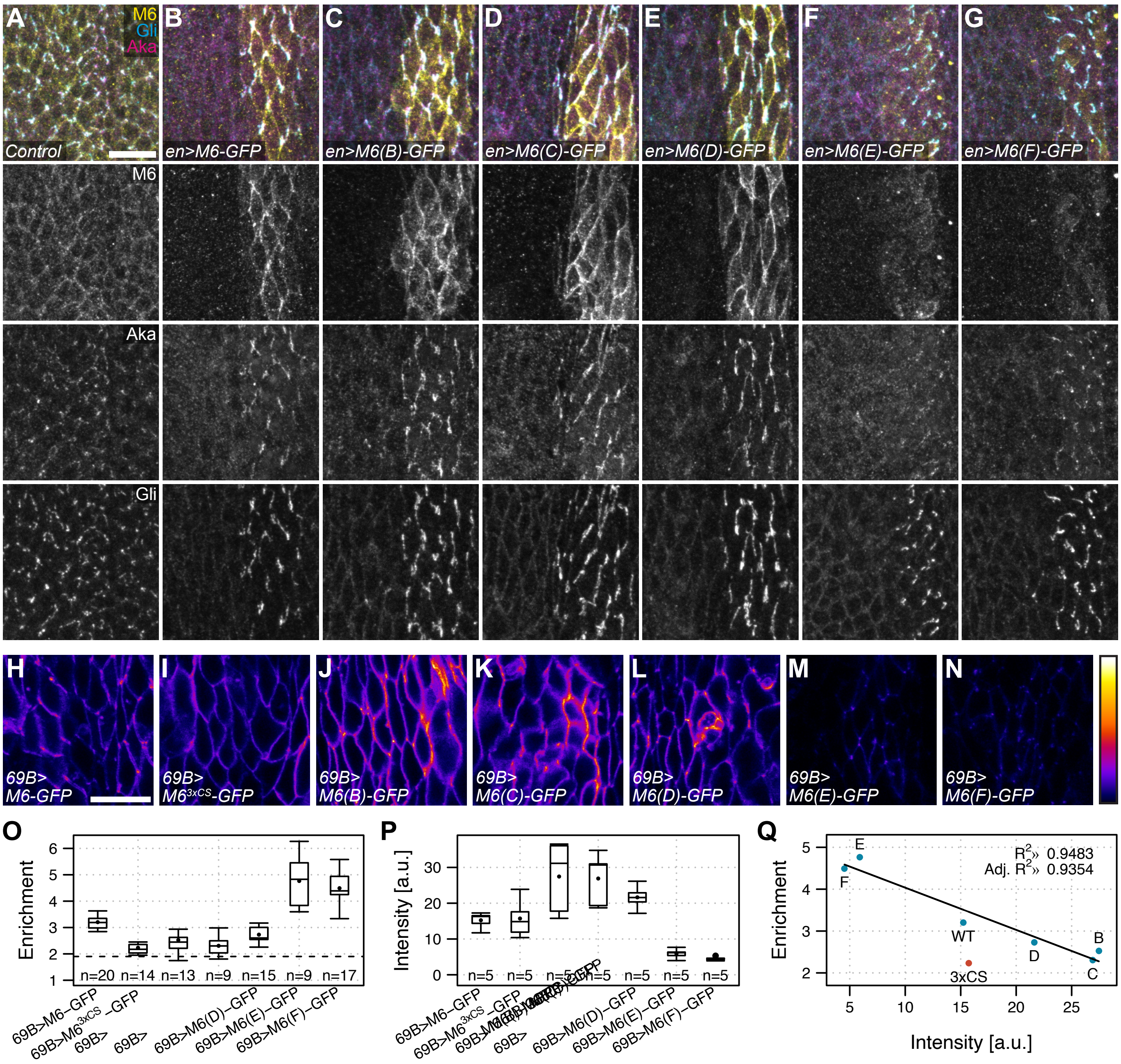

Fig. S2 (related to Fig. 1 and Fig. 3): All M6 isoforms support TCJ formation.

(A–G) *En face* view of lateral epidermis in control (*y w*; A) and in *M6*-deficient (*M6^{MB02608}/Df(3L)BSC419*; B-G) embryos (stage 15) expressing UAS-M6-GFP (B), UAS-M6(B)-GFP (C), UAS-M6(C)-GFP (D), UAS-M6(D)-GFP (E), UAS-M6(E)-GFP (F), or UAS-M6(F)-GFP (G) in epidermal stripes under control of *en*-Gal4. Embryos were fixed and immunostained against Aka, M6, and Gli. Note that all M6 isoforms rescue TCJ localization of Aka and Gli. M6 Isoforms E and F show low expression levels and rescue Aka localization only partially.

(H–N) *En face* view of lateral epidermis of living embryos (stage 15) expressing the indicated M6-GFP constructs under control of *69B*-Gal4. Images were taken with identical settings.

(O, P) Enrichment (O; measured as in Fig. 3) and fluorescence intensities (P) for each M6-GFP construct. M6 isoforms B, C, and D are strongly expressed whereas isoforms E and F are weakly expressed. Note that M6-GFP and $M6^{3xCS}$-GFP show similar expression levels.

(Q) Linear regression modelling vertex enrichment as a function of fluorescence intensity. Only blue data points were taken into consideration and follow a linear relationship. $M6^{3xCS}$-GFP (red) is shown for comparison and does not follow this linear relationship. (Adj.) R^2 denotes (adjusted) coefficient of determination for the model.

Scale bars: (A-G, H-N), 10 μm.

Journal of Cell Science • Supplementary information

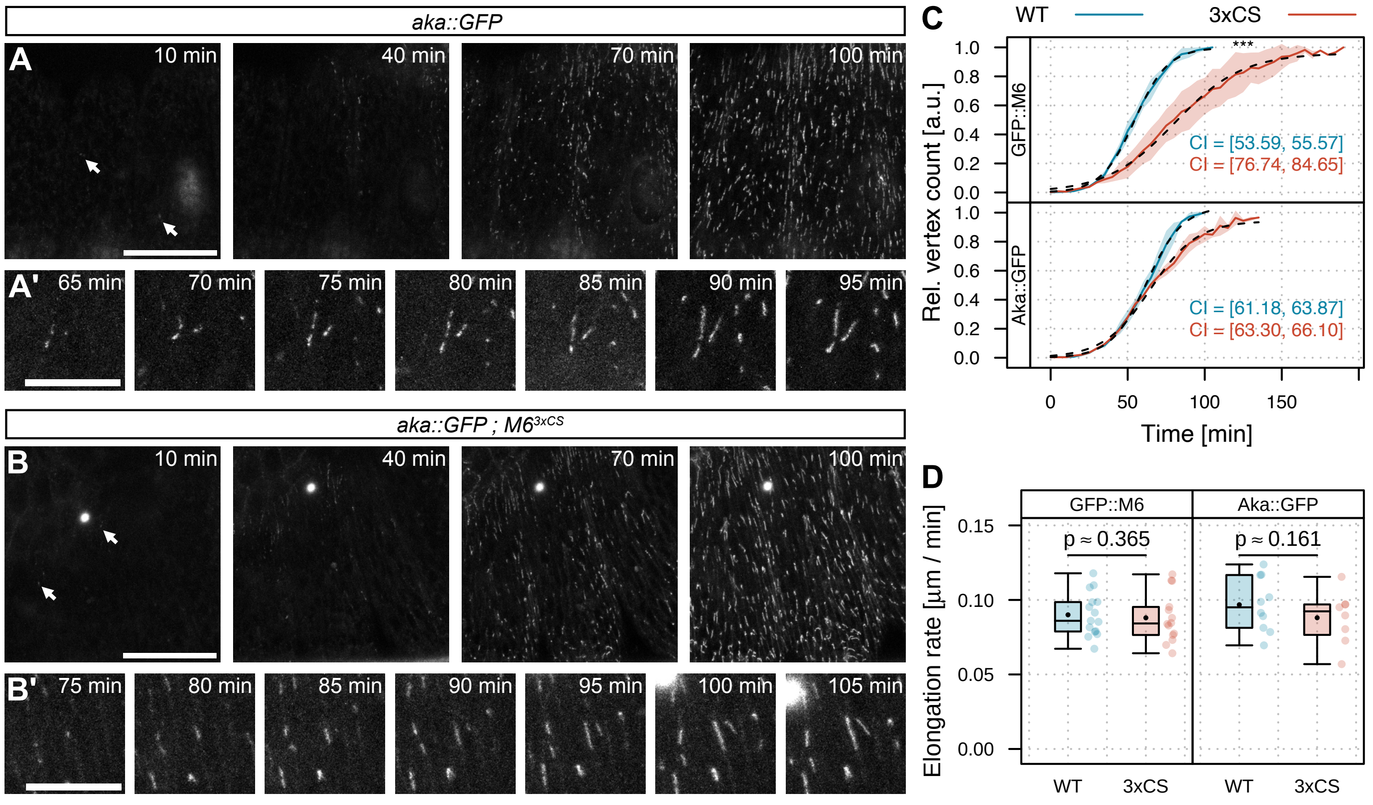

Fig. S3 (related to Fig. 4): Lack of M6 palmitoylation leads to delayed vertex accumulation of Aka::GFP but does not affect the rate of extension along vertices.

(A, B) *En face* view of dorso-lateral epidermis in living embryos (stage 13) expressing Aka::GFP in wild-type control (A) or homozygous *M6*^{3xCS} mutant (B) background. White arrows indicate first appearance of Aka::GFP clusters at vertices. Maximal-intensity projections are shown.

(A', B') Close-up showing extension of Aka::GFP signals along vertices in control (A') or *M6*^{3xCS} (B') embryos. Time is indicated. Rate of extension was quantified in (D).

(C) Quantification of vertex accumulation of Aka::GFP. Data for GFP::M6 (as in Fig. 4) is shown for comparison. The number of GFP-positive vertices was determined at each timepoint and normalized to the maximum number of GFP-positive vertices at the end of each movie. Note that GFP::M6 and Aka::GFP accumulate at most vertices throughout the epidermis within 100 min in wild-type (blue) embryos, whereas vertex accumulation is significantly delayed in palmitoylation-deficient *M6*^{3xCS} (red) embryos. Dashed lines indicate fitted logistic models. Confidence intervals (CI; upper and lower limit) for time of half-maximal accumulation are indicated. n=2 embryos per genotype for Aka::GFP, n=3 embryos per genotype for GFP::M6.

(D) Quantification of extension rate of GFP::M6 and Aka::GFP along vertices in control (blue) and *M6*^{3xCS} (red) embryos. There is no significant difference in elongation rate. 15 vertices in three embryos (GFP::M6) and 10 vertices in two embryos (Aka::GFP) were analyzed. One-sided unpaired *t*-test.

Scale bars: (A,B), 50 µm; (A', B'), 2 µm.

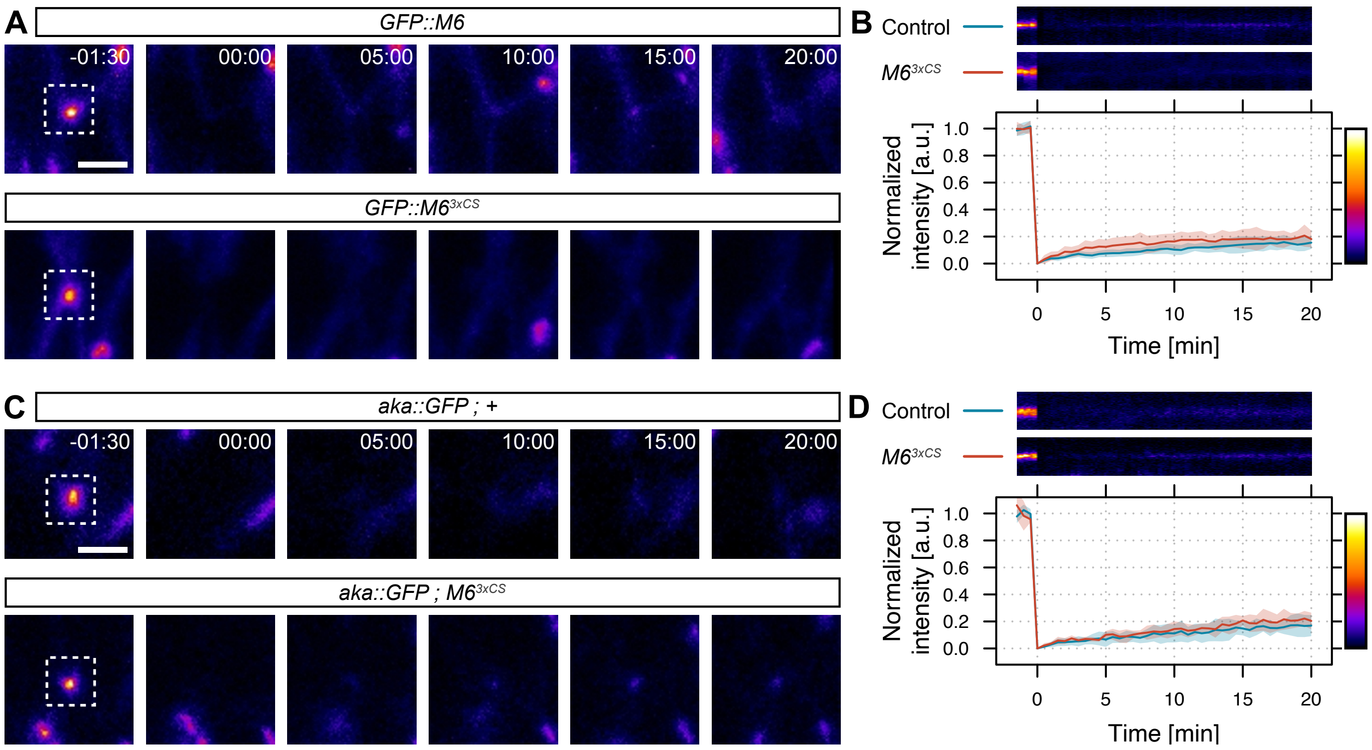

Fig. S4 (related to Fig. 4): Palmitoylation does not significantly alter maintenance or mobility of M6 at TCJs.

(A) Fluorescence recovery after photobleaching (FRAP) experiments in embryos (stage 15) homozygous for endogenous *GFP::M6* (control; top) or palmitoylation-deficient *GFP::M6*^{3xCS} (bottom). Time (min:sec) is indicated.

(B) Kymographs of series shown in (A) and quantification of fluorescence recovery indicate similar mobility of GFP::M6 (control; blue) and GFP::M6^{3xCS} (red).

(C) FRAP experiments in embryos (stage 15) expressing endogenous Aka::GFP in control (top) or in palmitoylation-deficient *M6*^{3xCS} embryos (bottom). Time (min:sec) is indicated.

(D) Kymographs of series shown in (C) and quantification of fluorescence recovery indicate similar mobility of Aka::GFP in control (blue) and in *M6*^{3xCS} (red) embryos.

Scale bars: (A,C), 2 µm.

Journal of Cell Science • Supplementary information

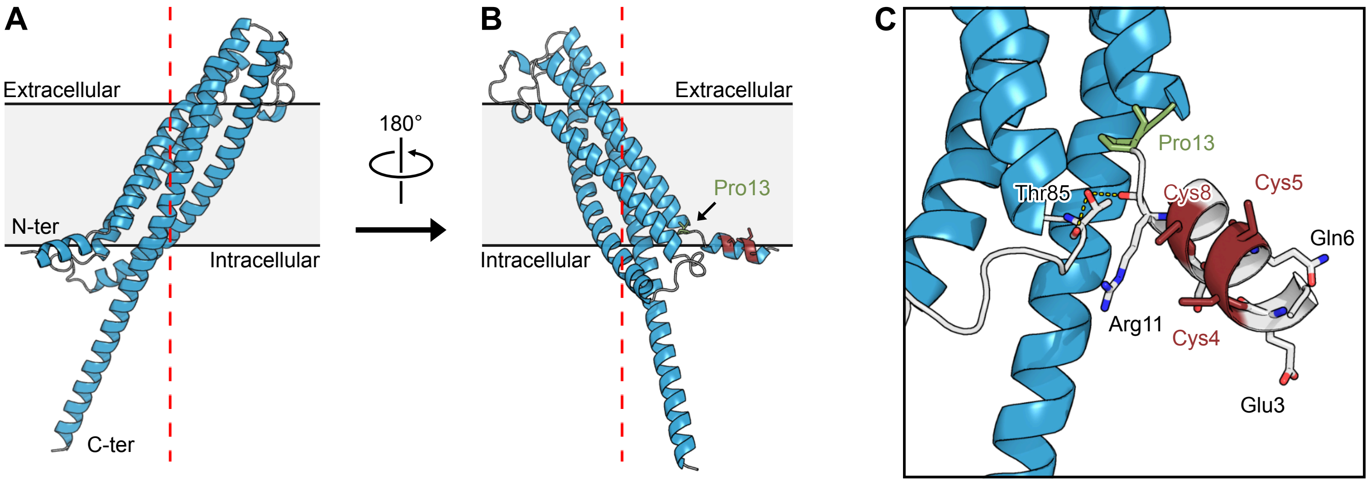

Fig. S5 (related to Fig. 2): Predicted orientation of M6 protein in the plasma membrane.

(A) Structure prediction of M6-D from AlphaFold (ID: AF-Q9NGC6-F1) was analyzed using the PPM3 webservice to determine orientation of the protein in the membrane. The protein displays a coiled-coil structure and is tilted 31 degrees with respect to the membrane-perpendicular line (red dashed line).

(B) As in (A) but rotated by 180°. The three conserved juxtamembrane cysteines near the N-terminus are highlighted in red. A proline at position 13 (green) induces a kink that bends the N-terminus towards the plasma membrane.

(C) Close-up view of the palmitoylated juxtamembrane region. Cysteines are shown in dark red, proline is shown in green. Arg11 engages in a hydrogen bond with Thr85 in the cytosolic loop. Charged or polar side chains are shown.

Journal of Cell Science • Supplementary information

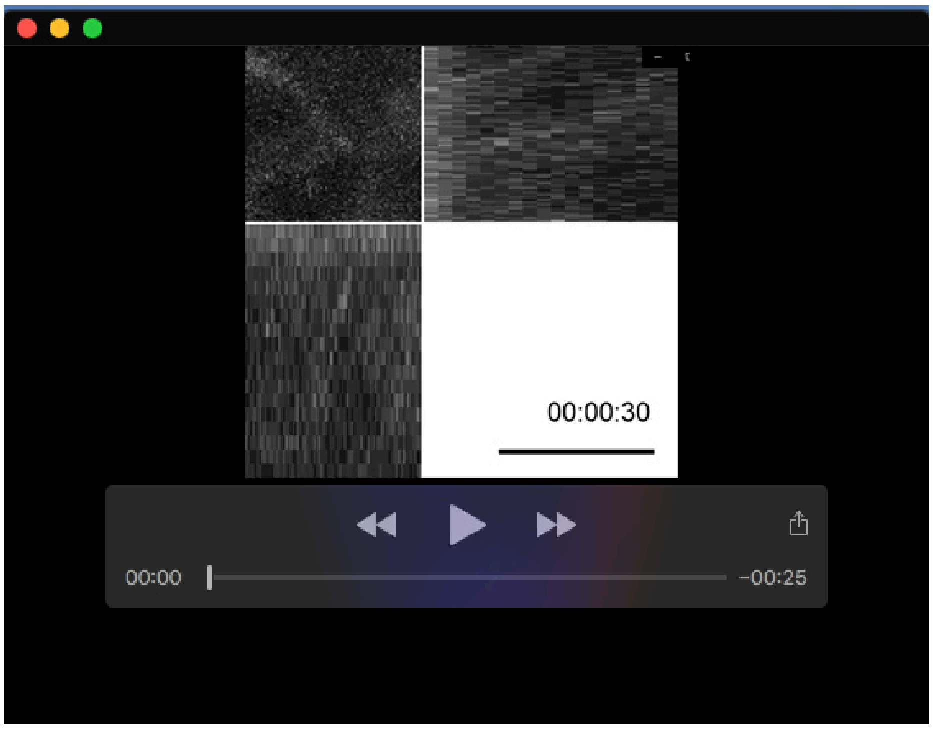

Movie 1 (related to Fig. 4). Extension of GFP::M6 along a vertex during TCJ formation.

En face view (top left) and orthogonal views of a single vertex accumulating GFP::M6 in lateral epidermis of stage 13 embryo. Note that GFP::M6 initially accumulates in a single spot at the apical tip of the vertex and that GFP::M6 signal subsequently extends basalwards along the vertex. See also Wittek et al. (2020). Time (h : min : sec) is indicated.

Scale bar: 5µm.

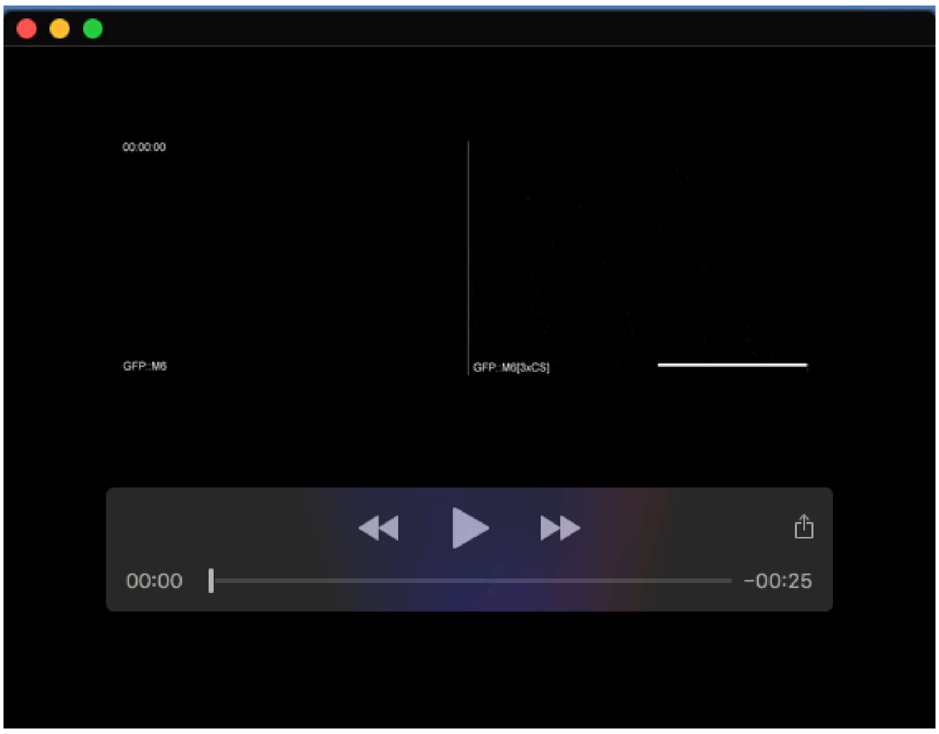

Movie 2 (related to Fig. 4). Time-lapse movies of M6 accumulation at epidermal cell vertices.

Dorso-lateral view of epidermis in stage 13 embryos homozygous for endogenous GFP::M6 (left) or GFP::M6^{3xCS} (right). Movies were aligned to the timepoint (t=30 min) that shows first enrichment of GFP signals at 5% of the final number of vertices. Note the delay in vertex accumulation of GFP::M6^{3xCS} compared to GFP::M6. The movie of the *GFP::M6* control embryo ends at t=100 min. Time (min) is indicated.

Scale bar: 50µm.

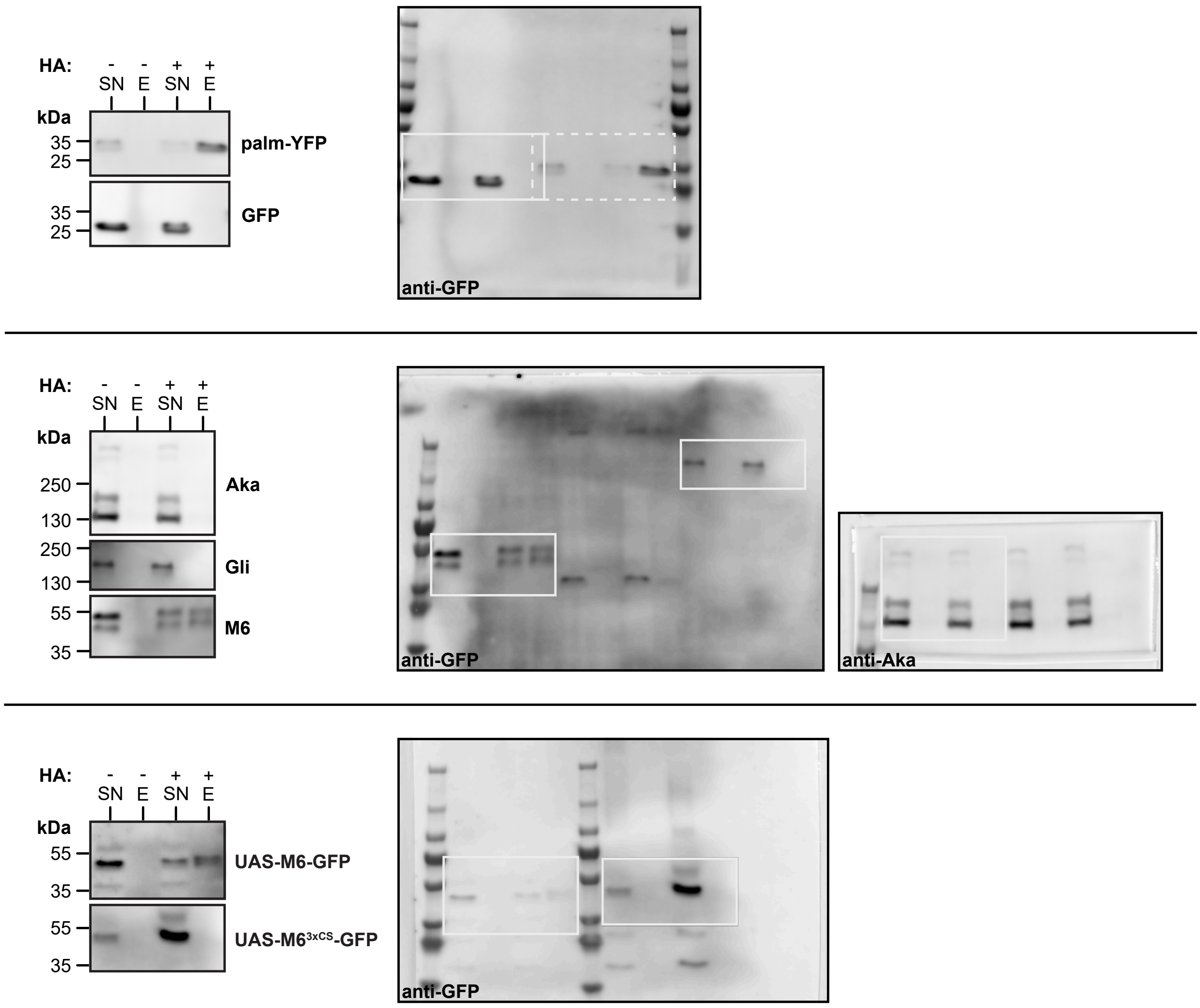

Fig. S6. Blot transparency related to Fig. 2.

Transparency blots are shown as a merge of the raw blot (chemiluminescence) and the marker (colorimetric). Corresponding regions in the processed blots are highlighted by white rectangles. Dashed lines were used to avoid ambiguity with overlapping regions.

Journal of Cell Science • Supplementary information

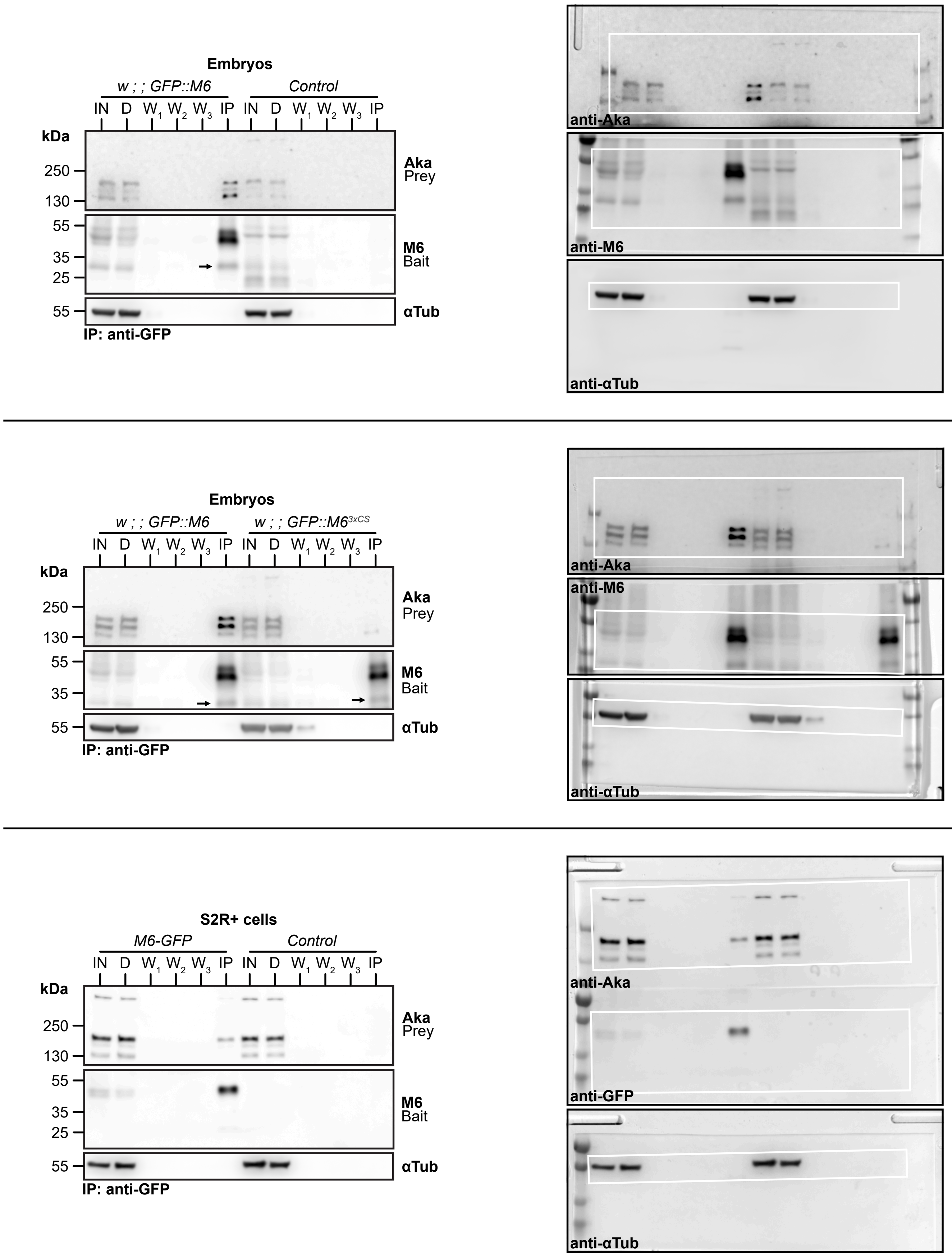

Fig. S6. Blot transparency related to Fig. 5B–D.

Transparency blots are shown as a merge of the raw blot (chemiluminescence) and the marker (colorimetric). Corresponding regions in the processed blots are highlighted by white rectangles. The size marker of the first αTub staining is missing.

Journal of Cell Science • Supplementary information

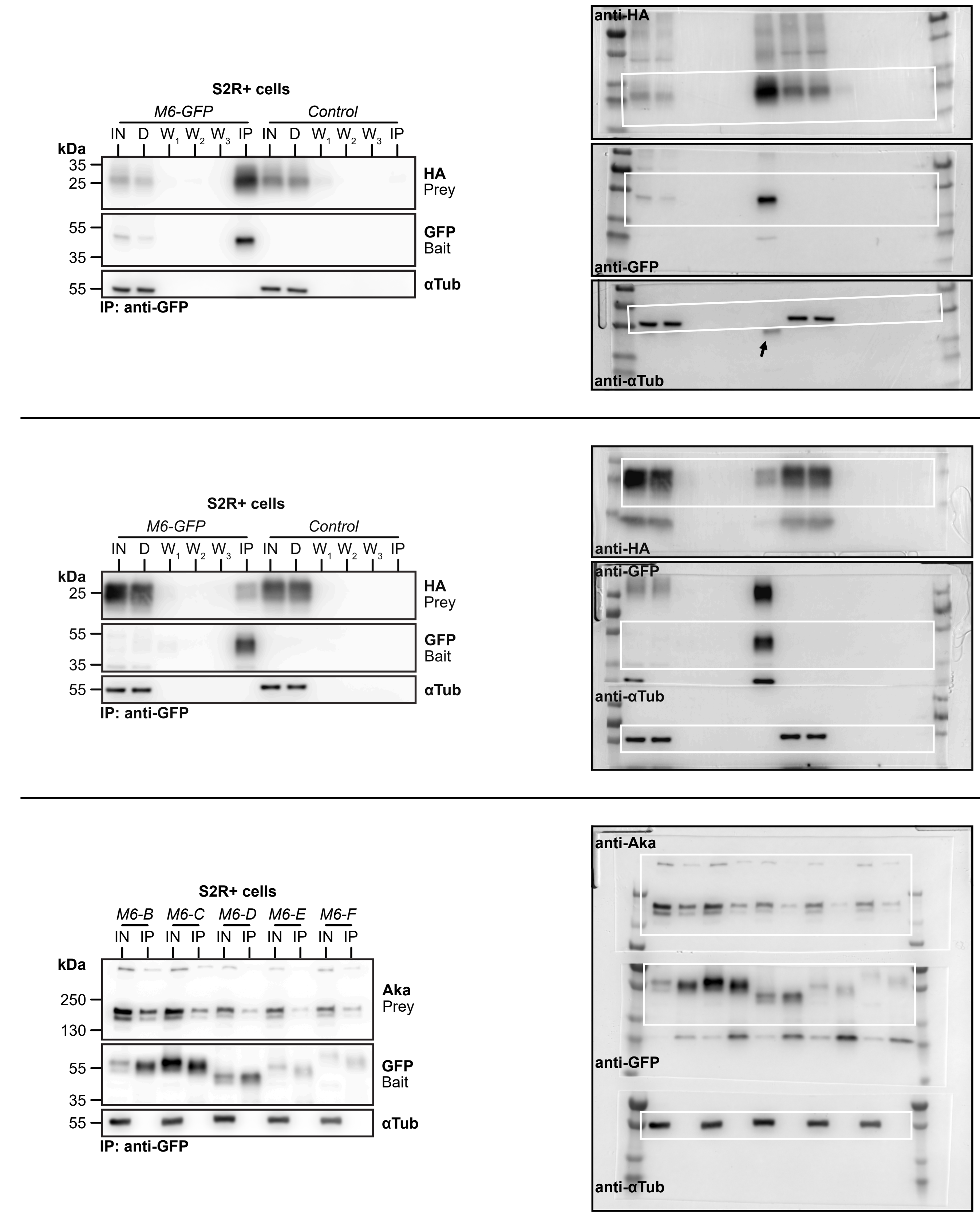

Fig. S6. Blot transparency related to Fig. 5E–G.

Transparency blots are shown as a merge of the raw blot (chemiluminescence) and the marker (colorimetric). Corresponding regions in the processed blots are highlighted by white rectangles. A prominent band in the center of the first αTub staining is visible (black arrow). This band results from incomplete stripping of the membrane after staining against GFP (shown above).

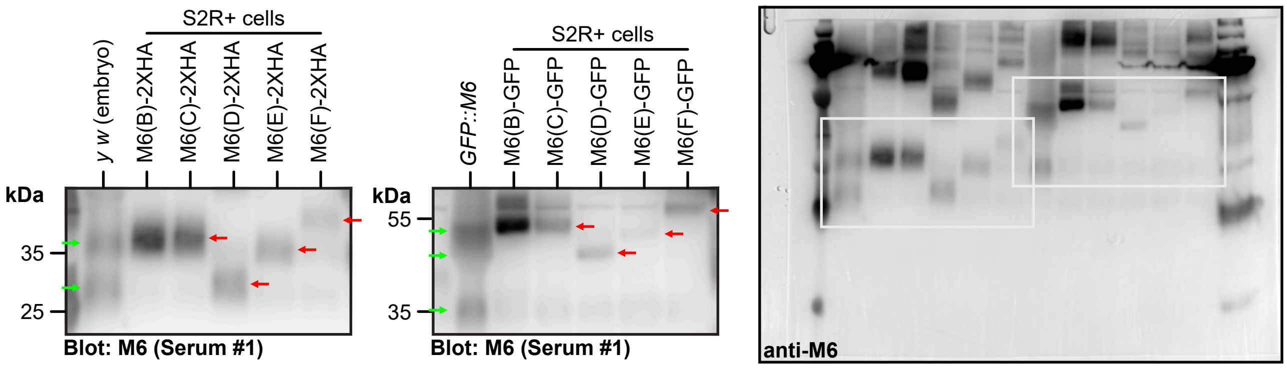

Fig. S6. Blot transparency related to Fig. S1B, C.

Transparency blots are shown as a merge of the raw blot (chemiluminescence) and the marker (colorimetric). Corresponding regions in the processed blots are highlighted by white rectangles.
