# Supplementary material for: Palmitoylation of proteolipid protein M6 promotes tricellular junction assembly in epithelia of Drosophila
Source: J Cell Sci. 2024 Mar 20;137(6):jcs261916. doi: 10.1242/jcs.261916 (PMC11698045; doi:10.1242/jcs.261916)
Supplement: Supplementary file 1 [file joces-137-261916_review_history.pdf]

Palmitoylation of proteolipid protein M6 promotes tricellular junction assembly in epithelia of Drosophila

Raphael Schleutker and Stefan Luschnig
DOI: 10.1242/jcs.261916

Editor: Michael Way

Review timeline

Submission to Review Commons:	7 September 2023
Submission to Journal of Cell Science:	18 December 2023
Editorial decision:	21 December 2023
First revision received:	2 February 2024
Accepted:	2 February 2024

Reviewer 1

Evidence, reproducibility and clarity

The epithelial diffusion barrier in triangular junctions is initially formed by a protein complex of Aka, Gli and M6. Aka and M6 act upstream of Gli. GPM6a, the vertebrate homolog of M6 is palmitoylated, whose functional implications have not thoroughly been analyzed, yet. It order to better define the function of M6 and especially the role of the palmitoyl moity, the authors conducted a genetic analysis of M6 in Drosophila embryos.

- They first establish a genetic system with defined mutants (deletion and CS mutants which are not palmitoylated), tagged protein at the genetic locus and an quantitative assay for protein enrichment at triangular junctions. Secondly they provide biochemical evidence that M6 is palmitoylated at a cluster of three conserved cysteine residues in vivo. With a palmitoylation-deficient mutant, thirdly the authors investigate the function of the palmitoyl moiety for protein localization at triangular junctions and complex formation with the other proteins at triangular junctions.
The authors reveal a quantitative function of the palmitoyl moiety at triangular junctions with respect to enrichment and initial accumulation but not for later functions during growth of triangular junctions. The lower enrichment of the non-palmitoylated M6 mutant are sufficient for recruitment of Aka and Gli. Importantly, reducing Aka in combination with the non- palmitoylated mutant leads to a strong phenotype with respect to Gli localization and and leads to a genetically synthetic embryonic lethality. Fourthly, on a molecular level, the palmitoyl residue mediates binding to Aka but is not required for di/oligomerization of M6 itself as shown by immunoprecipitation from embryonic lysates.

- Though the function of M6 acting together with Aka and Gli has been demonstrated previously, molecular details of the interactions and targeting of the proteins to triangular junctions have remained unclear. Similarly, although palmitoylation of the vertebrate homologue has been previously demonstrated, its functional implications have not been investigated in a physiological context with stringent genetics. The current study provides convincing data about the role of the palmitoylated moiety of M6. Importantly, the authors manage to differentiate a function of the palmitoyl residue in initial accumulation of M6 at triangular junctions versus maintenance. Also the authors manage to reveal an essential function of M6 palmitoylation when the dose of Aka is reduced. In summary, the study provides novel and interesting insights into the detailed molecular requirements of epithelial barrier formation. Although the quality of the data and analysis provides

© 2024. Published by The Company of Biologists under the terms of the Creative Commons Attribution License (https://creativecommons.org/licenses/by/4.0/).

an argument for publication on its own, it may be noted that similar mechanisms may underlie barrier formation at triangular junction in vertebrates given the conservation of the protein components.

Minor comments:

1. L100: it is stated that "... is not detectable on other known TCJ components". What about Angulin-1, which is palmitoylated?

2. L122 In my understanding all M6 isoforms contain an element which is sufficient. Not "required".

3. L336. The allele designation "DeltaPalm" is misleading. A designation like "3xCS" would be more better because three defined cysteine residues are mutated.

4. L329 A reference to FLYBASE is missing. Similarly not reference to stock centers are provides. To document the importance of the community services it is essential that their services are properly cited in a way that can be automatically tracked, e. g. by a literature citation.

Significance

In summary, the study provides novel and interesting insights into the detailed molecular requirements of epithelial barrier formation. Although the quality of the data and analysis provides an argument for publication on its own, it may be noted that similar mechanisms may underlie barrier formation at triangular junction in vertebrates given the conservation of the protein components.

Reviewer 2

Evidence, reproducibility and clarity

Summary:

This article by Raphael Schleutker and Stefan Luschnig addresses the importance of S-palmitoylation of the proteolipid protein M6, one of the three components of tricellular septate junction along with Anakonda and Giotactin, in the assembly of tricellular junctions using Drosophila embryo as a model system. Using a combination of state-of-the-art genome engineering, live imaging and biochemistry, the authors demonstrated that M6 is palmitoylated in vivo, elegantly identified the cysteine residue that is palmitoylated, showed that this modification is essential for interaction with Anakonda and provided convincing evidence that palmytoylation is required for the initial assembly of tricellular junctions.

Major comments:

- Are the claims and the conclusions supported by the data or do they require additional experiments or analyses to support them?
The claims are largely supported by the very high quality data assembled in this article. I have just a few concerns that can be resolved by modifying the text or, optionally, by carrying out additional experiments:

1. in the summary (lane 40) and all along the article it is stated that 'Abolishing M6 palmitoylation leads to delayed accumulation of M6 and Aka at vertices but does not affect the rate of TCJ growth or mobility of M6 or Aka.'
However, whilst the data presented convincingly demonstrate the delayed localization of GFP::M6 delta Palm at TCJ, that of Aka at TCJ is not shown. Although I think this is a reasonable hypothesis, without showing Aka localization, this claim is too strong and should be toned down, or better (optional) show the dynamics of Aka localization.
Lanes 184 and 197 'indicating that effcient TCJ formation depends on M6 palmitoylation.' TCJ formation is not assessed here, what is measured is the localization of M6 at vertex. I suggest to amend the text accordingly.

© 2024. Published by The Company of Biologists under the terms of the Creative Commons Attribution License (https://creativecommons.org/licenses/by/4.0/).

2. Fig. 5C and lane 292' Lack of M6 palmitoylation reduces, although it does not completely abolish, the interaction with Aka,....
In Fig. 5C, GFP::M6 efficiently co-precipitates three forms of Aka with different molecular weights. The two upper bands are highly enriched in the GFP::M6 coIp. In contrast, GFP::M6 delta Palm seems to coIp only the low molecular weight form of Aka. Could the authors explain what the three forms of Aka are, and provide potential explanations or interpretations of this result?

- Please request additional experiments only if they are essential for the conclusions. Alternatively, ask the authors to qualify their claims as preliminary or speculative, or to remove them altogether.

- Are the data and the methods presented in such a way that they can be reproduced? The data are of very high quality and the methods sufficiently described (with appropriate references where necessary) and presented in such a way that they can be reproduced.

- Are the experiments adequately replicated and statistical analysis adequate?
Although the microscopy data are perfectly quantified and the appropriate statistical tests are used, unless I am mistaken, the number of replicates and the number of independent experiments carried out in biochemistry (Fig. 2 and Fig. 5) are not indicated.

Minor comments:

- Specific experimental issues that are easily addressable.

1. The localization of M6 on living specimens, GFP::M6 enriched at the tricellular junction, differs from the localization of M6 detected by anti-M6 on fixed samples, i.e. M6 homogenous distributed at the bicellular junction, no enrichment at the tricellular junction. Please comment and possibly explain the reason for the difference in localisation. Is the anti-GFP staining on the GFP::M6 sample restricted to the bicellular junction without apparent TCJ enrichment?

2. In Fig. S2, isoforms E and F are expressed at low level but fully rescue Gli localization but not Aka. These results are somewhat surprising if Gli localization relies on Aka and M6 localization at TCJ. Is localization of M6 at TCJ important or is it the expression of M6 that matters? Would it be possible to compare the expression levels of the different isoforms?.

3. lane 118 'Notably, vertex enrichment varied between M6-GFP isoforms and was inversely proportional to overall signal intensity, suggesting that saturation effects upon overexpression impede vertex enrichment. Consistent with this notion, endogenous
GFP::M6CA06602 showed higher vertex enrichment (7.8-fold; Fig. 1E, L) than the individual overexpressed isoforms.'
To conclude that all isoforms contain the elements for vertex localization, it would be interesting to provide the level of expression (signal intensity) for all M6 isoform as well as M6deltaPAlm-GFP to appreciate the threshold above which saturation is achieved? Or better (optional) to express the different isoforms in a M6 mutant background.
Could the authors exclude the possibility that the position of the GFP moiety affect the localization of M6 at TCJ?

4. lanes 187 '...in a single spot that subsequently extends basally with a speed of 0.09 µm/min '
The images are presumably projections along the apical basal axis, so it is difficult to appreciate the apical to basal extension, perhaps an orthogonal section would help.

- Are prior studies referenced appropriately? yes

- Are the text and figures clear and accurate? yes

Significance

Provide contextual information to readers (editors and researchers) about the novelty of the study, its value for the field and the communities that might be interested.

© 2024. Published by The Company of Biologists under the terms of the Creative Commons Attribution License (https://creativecommons.org/licenses/by/4.0/).

The following aspects are important:

Tricellular junctions are hot spots integrating mechanical and chemical inputs that are essential to ensure epithelia homeostasis. It is therefore essential to understand how the components of tricellular junctions are located and assembled to form functional tricellular junctions. The authors brilliantly demonstrate the key role of S-palmitoylation in M6 localization and ability to interact with Aka in vivo. The fact that the role of palmitoylation appears to be conserved for the assembly of vertebrate TCJs, made up of components that are not conserved throughout evolution, indicates a fundamental function of palmitoylation in protein-protein interactions at the level of TCJs and in their vesicular trafficking. As palmitoylation is reversible, this work also raises the question of how palmitoylation is regulated in time and space to ensure the plasticity of TCJs in developing epithelia.

- *General assessment:* provide a summary of the strengths and limitations of the study. What are the strongest and most important aspects? What aspects of the study should be improved or could be developed?

- *Advance:* compare the study to the closest related results in the literature or highlight results reported for the first time to your knowledge; does the study extend the knowledge in the field and in which way? Describe the nature of the advance and the resulting insights (for example: conceptual, technical, clinical, mechanistic, functional,...).

This study follows on from that showing the role of Angulin1 palmitoylation in its localization to tricellular junctions in vertebrates. The present study demonstrates the conserved nature of the role of this post-translational modification in the assembly of complex membrane structures essential for epithelial homeostasis. In addition, it demonstrates the dynamic nature and temporality of the role of palmytoylation in the early stages of recruitment of M6 to the vertex, opening up numerous hypotheses for future work at the conceptual and fonctional levels, elegantly presented in the discssion.

- *Audience:* describe the type of audience ("specialized", "broad", "basic research", "translational/clinical", etc...) that will be interested or influenced by this research; how will this research be used by others; will it be of interest beyond the specific field?

I believe this article is dedicated to a rather broad audience. Although this article may at first appear to be aimed at specialists, the findings go beyond the interest in tricellular junctions in Drosophila, since the role of palmytoylation of tricellular components appears to be conserved in vertebrates. In addition, this study will have an impact on the overall cell biology community, including membrane trafficking and the role of lipid modification additions on the subcellular dynamics of transmembrane proteins in a physiological context.

- Please define your field of expertise with a few keywords to help the authors contextualize your point of view. Indicate if there are any parts of the paper that you do not have sufficient expertise to evaluate.
I am a developmental cell biologist, with an expertise in epithelial junctions and epithelial tissue homeostasis, using vertebrate and invertebrate model systems.

Author response to reviewers' comments

We would like to thank the referees for their thoughtful comments on our manuscript. We appreciate that both reviewers pointed out the quality of our work, as well as the significance and impact of the findings. Our point-by-point responses to the reviewers´ comments are highlighted in blue.

Reviewer #1 (Evidence, reproducibility and clarity (Required)):
The epithelial diffusion barrier in triangular junctions is initially formed by a protein complex of

© 2024. Published by The Company of Biologists under the terms of the Creative Commons Attribution License (https://creativecommons.org/licenses/by/4.0/).

Aka, Gli and M6. Aka and M6 act upstream of Gli. GPM6a, the vertebrate homolog of M6 is palmitoylated, whose functional implications have not thoroughly been analyzed, yet. It order to better define the function of M6 and especially the role of the palmitoyl moity, the authors conducted a genetic analysis of M6 in Drosophila embryos.

They first establish a genetic system with defined mutants (deletion and CS mutants which are not palmitoylated), tagged protein at the genetic locus and an quantitative assay for protein enrichment at triangular junctions. Secondly they provide biochemical evidence that M6 is palmitoylated at a cluster of three conserved cysteine residues in vivo. With a palmitoylation-deficient mutant, thirdly the authors investigate the function of the palmitoyl moiety for protein localization at triangular junctions and complex formation with the other proteins at triangular junctions.
The authors reveal a quantitative function of the palmitoyl moiety at triangular junctions with respect to enrichment and initial accumulation but not for later functions during growth of triangular junctions. The lower enrichment of the non-palmitoylated M6 mutant are sufficient for recruitment of Aka and Gli. Importantly, reducing Aka in combination with the non- palmitoylated mutant leads to a strong phenotype with respect to Gli localization and and leads to a genetically synthetic embryonic lethality. Fourthly, on a molecular level, the palmitoyl residue mediates binding to Aka but is not required for di/oligomerization of M6 itself as shown by immunoprecipitation from embryonic lysates.

Though the function of M6 acting together with Aka and Gli has been demonstrated previously, molecular details of the interactions and targeting of the proteins to triangular junctions have remained unclear. Similarly, although palmitoylation of the vertebrate homologue has been previously demonstrated, its functional implications have not been investigated in a physiological context with stringent genetics. The current study provides convincing data about the role of the palmitoylated moiety of M6. Importantly, the authors manage to differentiate a function of the palmitoyl residue in initial accumulation of M6 at triangular junctions versus maintenance. Also the authors manage to reveal an essential function of M6 palmitoylation when the dose of Aka is reduced. In summary, the study provides novel and interesting insights into the detailed molecular requirements of epithelial barrier formation. Although the quality of the data and analysis provides an argument for publication on its own, it may be noted that similar mechanisms may underlie barrier formation at triangular junction in vertebrates given the conservation of the protein components.

Minor comments

L100: it is stated that "... is not detectable on other known TCJ components". What about Angulin-1, which is palmitoylated?

We apologize for the misleading sentence. The passage refers only to Drosophila TCJ proteins as shown in Figure 2C. We rephrased the sentence as follows:
"… is not detectable on other known TCJ components in *Drosophila.*"

L122 In my understanding all M6 isoforms contain an element which is sufficient. Not "required".

Thank you for pointing out this logical mistake. We changed the statement as follows: "We conclude that all M6 isoforms contain the element(s) sufficient for vertex localization."

L336. The allele designation "DeltaPalm" is misleading. A designation like "3xCS" would be more better because three defined cysteine residues are mutated.

We agree and we changed the allele designation to "*3xCS*", as suggested by the reviewer.

L329 A reference to FLYBASE is missing. Similarly not reference to stock centers are provides. To document the importance of the community services it is essential that their services are properly cited in a way that can be automatically tracked, e. g. by a literature citation.

© 2024. Published by The Company of Biologists under the terms of the Creative Commons Attribution License (https://creativecommons.org/licenses/by/4.0/).

We appreciate the comment, and we agree about the importance of properly acknowledging community services. We added a reference to FlyBase (Thurmond *et al*. (2019) Nucleic Acids Research 47, D759-65) and the following statement to the acknowledgement section:

"We acknowledge service by the Developmental Studies Hybridoma Bank, created by the NICHD of the NIH and maintained at the University of Iowa, and by the Bloomington Drosophila Stock Center (NIH P40OD018537)."

Reviewer #2 (Evidence, reproducibility and clarity (Required)):

Summary:

This article by Raphael Schleutker and Stefan Luschnig addresses the importance of S-palmitoylation of the proteolipid protein M6, one of the three components of tricellular septate junction along with Anakonda and Giotactin, in the assembly of tricellular junctions using Drosophila embryo as a model system. Using a combination of state-of-the-art genome engineering, live imaging and biochemistry, the authors demonstrated that M6 is palmitoylated in vivo, elegantly identified the cysteine residue that is palmitoylated, showed that this modification is essential for interaction with Anakonda and provided convincing evidence that palmytoylation is required for the initial assembly of tricellular junctions.

Major comments

- Are the claims and the conclusions supported by the data or do they require additional experiments or analyses to support them?

The claims are largely supported by the very high quality data assembled in this article. I have just a few concerns that can be resolved by modifying the text or, optionally, by carrying out additional experiments:

1- in the summary (lane 40) and all along the article it is stated that 'Abolishing M6 palmitoylation leads to delayed accumulation of M6 and Aka at vertices but does not affect the rate of TCJ growth or mobility of M6 or Aka.' However, whilst the data presented convincingly demonstrate the delayed localization of GFP::M6 delta Palm at TCJ, that of Aka at TCJ is not shown. Although I think this is a reasonable hypothesis, without showing Aka localization, this claim is too strong and should be toned down, or better (optional) show the dynamics of Aka localization.

We agree with the reviewer. We showed that Aka is able to localize at TCJs in the absence of M6 palmitoylation (Fig. 3N, P), but we did not provide data on the dynamics of Aka localization. We are currently performing experiments (time-lapse movies analogous to those shown for GFP::M6 in Fig. 4) to analyze the dynamics of Aka::GFP localization in control and in $M6^{3xCS}$ embryos. We will include the results in the revised manuscript.

Lanes 184 and 197 'indicating that effcient TCJ formation depends on M6 palmitoylation.' TCJ formation is not assessed here, what is measured is the localization of M6 at vertex. I suggest to amend the text accordingly.

We agree that the presented results address the localization of M6 rather than the process of TCJ formation. We therefore rephrased the corresponding statements as follows:

L184: "Thus, although palmitoylation is not essential for vertex localization of M6, it promotes transport to or maintenance of M6 at vertices."

L197: "... indicating that efficient TCJ localization of M6 depends on palmitoylation."

2- Fig. 5C and lane 292' Lack of M6 palmitoylation reduces, although it does not completely abolish,

© 2024. Published by The Company of Biologists under the terms of the Creative Commons Attribution License (https://creativecommons.org/licenses/by/4.0/).

the interaction with Aka, ' In Fig. 5C, GFP::M6 efficiently co-precipitates three forms of Aka with different molecular weights. The two upper bands are highly enriched in the GFP::M6 coIp. In contrast, GFP::M6 delta Palm seems to coIp only the low molecular weight form of Aka. Could the authors explain what the three forms of Aka are, and provide potential explanations or interpretations of this result?

We appreciate the comment. The ectodomain of Aka protein is proteolytically cleaved, as we showed previously (Byri *et al*. Dev Cell 2015). Antisera raised against a peptide in Aka´s cytoplasmic domain detect C-terminal fragments comprising the transmembrane domain and cytoplasmic tail as protein species of approximately 140 and 160 kDa, which correspond to the upper two bands that co-precipitate with GFP::M6. The occurrence of the third, lower molecular weight band of approx. 130 kDa that co-precipitates with palmitoylation-deficient GFP::M6(3CS) turned out to be variable, whereas the two upper (140 and 160 kDa) bands are always detectable. We are currently re-examining the identity of the 130 kDa band by probing lysates from wild-type and *aka*-deficient embryos. The results will be included in the revised manuscript.

- Please request additional experiments only if they are essential for the conclusions. Alternatively, ask the authors to qualify their claims as preliminary or speculative, or to remove them altogether.

- Are the data and the methods presented in such a way that they can be reproduced?
The data are of very high quality and the methods sufficiently described (with appropriate references where necessary) and presented in such a way that they can be reproduced.

- Are the experiments adequately replicated and statistical analysis adequate?

Although the microscopy data are perfectly quantified and the appropriate statistical tests are used, unless I am mistaken, the number of replicates and the number of independent experiments carried out in biochemistry (Fig. 2 and Fig. 5) are not indicated.

We amended the legends of Fig. 2 and Fig. 5 to indicate the number of independent repeats for the biochemistry experiments. Two independent repeats were carried out for each acyl- biotin-exchange experiment shown in Figure 2B, C,F. At least three independent experiments were performed for each IP experiment shown in Figure 5.

Minor comments:

- Specific experimental issues that are easily addressable.

1- The localization of M6 on living specimens, GFP::M6 enriched at the tricellular junction, differs from the localization of M6 detected by anti-M6 on fixed samples, i.e. M6 homogenous distributed at the bicellular junction, no enrichment at the tricellular junction. Please comment and possibly explain the reason for the difference in localisation. Is the anti-GFP staining on the GFP::M6 sample restricted to the bicellular junction without apparent TCJ enrichment?

The reviewer is correct on the different appearance of M6 protein distribution in live and fixed specimens, respectively. While we have no experimentally validated explanation for this observation, we speculate that vertex-localized M6 protein may not be efficiently recognized by the anti-M6 antiserum, possibly because the epitope may be inaccessible when M6 protein is incorporated into the TCJ protein complex. The difference does not seem to result from the fixation and staining procedure, as staining with anti-GFP antibodies reproduces the distribution of GFP::M6 seen in live embryos. We will add a close-up image to Supplemental Figure 1 to illustrate this point.

2- In Fig. S2, isoforms E and F are expressed at low level but fully rescue Gli localization but not Aka. These results are somewhat surprising if Gli localization relies on Aka and M6 localization at TCJ. Is localization of M6 at TCJ important or is it the expression of M6 that matters? Would it be possible to compare the expression levels of the different isoforms?.

© 2024. Published by The Company of Biologists under the terms of the Creative Commons Attribution License (https://creativecommons.org/licenses/by/4.0/).

Thank you for the careful observation and the thoughtful comment. We agree with the reviewer´s suggestion that the levels of M6 protein are likely to play an important role here. TCJ localization of M6 does not appear to be critical when overall levels of M6 at the plasma membrane are sufficiently high, as in the overexpression experiment referred to here (Fig. S2). M6 isoforms E and F show substantially lower expression levels (see below) compared to all other M6 isoforms, and do not fully rescue Aka localization. Albeit surprising, these results suggest that TCJ localization of Aka depends more strongly than that of Gli on the levels of M6. To substantiate this idea, we quantified the expression levels of M6 isoforms (see below).

3- lane 118 'Notably, vertex enrichment varied between M6-GFP isoforms and was inversely proportional to overall signal intensity, suggesting that saturation effects upon overexpression impede vertex enrichment. Consistent with this notion, endogenous GFP::M6CA06602 showed higher vertex enrichment (7.8-fold; Fig. 1E, L) than the individual overexpressed isoforms.' To conclude that all isoforms contain the elements for vertex localization, it would be interesting to provide the level of expression (signal intensity) for all M6 isoform as well as M6deltaPAlm-GFP to appreciate the threshold above which saturation is achieved? Or better (optional) to express the different isoforms in a M6 mutant background. Could the authors exclude the possibility that the position of the GFP moiety affect the localization of M6 at TCJ?

As suggested by the reviewer, we analyzed the expression levels of all individual GFP-tagged M6 isoforms as well as of palmitoylation-deficient M6(3xCS)-GFP in epidermal cells. For each construct, we compared the overall signal intensity to the factor of enrichment at TCJs, which revealed an inverse relationship (see figure below):

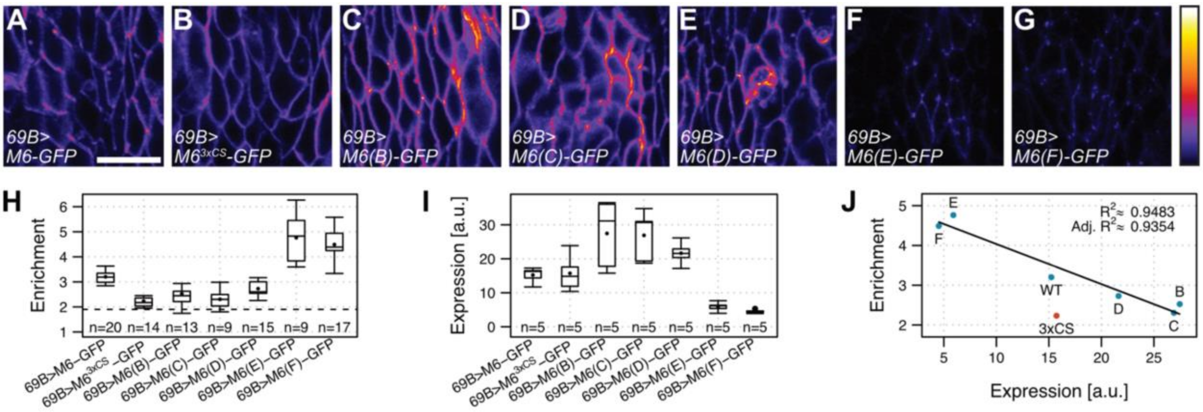

Importantly, this analysis also revealed that wild-type M6-GFP and palmitoylation-deficient M6-GFP(3xCS) are expressed at comparable levels, indicating that low vertex enrichment of M6-GFP(3xCS) is not a consequence of altered protein levels. We will add these new data to Figure S2, and we will discuss the results in the revised manuscript.

The reviewer raised the possibility that the position of the GFP tag may affect the localization of M6 at TCJs. We would like to note that in the overexpressed M6-GFP constructs the GFP tag is always at the C-terminus, which is invariant between M6 isoforms. Furthermore, we showed that both N- and C-terminally GFP-tagged M6 variants localize to TCJs, excluding the possibility that the position of the GFP tag has a major influence on the localization of GFP-M6 fusion proteins.

4- lanes 187 '...in a single spot that subsequently extends basally with a speed of 0.09 µm/min ' The images are presumably projections along the apical basal axis, so it is difficult to appreciate the apical to basal extension, perhaps an orthogonal section would help.

We appreciate the reviewer´s point that apical-to-basal extension of GFP::M6 accumulation along vertices is difficult to see in the images in Fig. 4A',B'. To better visualize the extension towards the basal side, we will add orthogonal sections and we will modify the close-up images in Fig. 4A’, B’ to

© 2024. Published by The Company of Biologists under the terms of the Creative Commons Attribution License (https://creativecommons.org/licenses/by/4.0/).

indicate vertex length using a color scale. Furthermore, we will include a supplemental movie that displays extension of GFP::M6 signals along the apicobasal axis.

-Are prior studies referenced appropriately?
yes

-Are the text and figures clear and accurate?
yes

Reviewer #2 (Significance (Required)):

Provide contextual information to readers (editors and researchers) about the novelty of the study, its value for the field and the communities that might be interested.

The following aspects are important:

Tricellular junctions are hot spots integrating mechanical and chemical inputs that are essential to ensure epithelia homeostasis. It is therefore essential to understand how the components of tricellular junctions are located and assembled to form functional tricellular junctions. The authors brilliantly demonstrate the key role of S-palmitoylation in M6 localization and ability to interact with Aka in vivo. The fact that the role of palmitoylation appears to be conserved for the assembly of vertebrate TCJs, made up of components that are not conserved throughout evolution, indicates a fundamental function of palmitoylation in protein-protein interactions at the level of TCJs and in their vesicular trafficking. As palmitoylation is reversible, this work also raises the question of how palmitoylation is regulated in time and space to ensure the plasticity of TCJs in developing epithelia.

- General assessment: provide a summary of the strengths and limitations of the study. What are the strongest and most important aspects? What aspects of the study should be improved or could be developed?

- Advance: compare the study to the closest related results in the literature or highlight results reported for the first time to your knowledge; does the study extend the knowledge in the field and in which way? Describe the nature of the advance and the resulting insights (for example: conceptual, technical, clinical, mechanistic, functional,...).

This study follows on from that showing the role of Angulin1 palmitoylation in its localization to tricellular junctions in vertebrates. The present study demonstrates the conserved nature of the role of this post-translational modification in the assembly of complex membrane structures essential for epithelial homeostasis. In addition, it demonstrates the dynamic nature and temporality of the role of palmytoylation in the early stages of recruitment of M6 to the vertex, opening up numerous hypotheses for future work at the conceptual and fonctional levels, elegantly presented in the discssion.

- Audience: describe the type of audience ("specialized", "broad", "basic research", "translational/clinical", etc...) that will be interested or influenced by this research; how will this research be used by others; will it be of interest beyond the specific field?

I believe this article is dedicated to a rather broad audience. Although this article may at first appear to be aimed at specialists, the findings go beyond the interest in tricellular junctions in Drosophila, since the role of palmytoylation of tricellular components appears to be conserved in vertebrates. In addition, this study will have an impact on the overall cell biology community, including membrane trafficking and the role of lipid modification additions on the subcellular dynamics of transmembrane proteins in a physiological context.

- Please define your field of expertise with a few keywords to help the authors contextualize your point of view. Indicate if there are any parts of the paper that you do not have sufficient expertise to evaluate.

I am a developmental cell biologist, with an expertise in epithelial junctions and epithelial tissue

© 2024. Published by The Company of Biologists under the terms of the Creative Commons Attribution License (https://creativecommons.org/licenses/by/4.0/).

homeostasis, using vertebrate and invertebrate model systems.

Original submission

First decision letter

MS ID#: JOCES/2023/261916

MS TITLE: Palmitoylation of proteolipid protein M6 promotes tricellular junction assembly in epithelia of Drosophila

AUTHORS: Raphael Schleutker and Stefan Luschnig
ARTICLE TYPE: Research Article

We have now reached a decision on the above manuscript.

To see a copy of this decision letter, please go to: https://submit-jcs.biologists.org and click on the 'Manuscripts with Decisions' queue in the Author Area. (Corresponding author only has access to reviews.)

Based on the reviewers reports from review commons and your response / revision plan, I think that the manuscript would be appropriate for publication in Journal of Cell Science.

Please ensure that you clearly highlight all changes made to the text in the revised manuscript when you re-submit. Please use a different colour text rather than 'Tracked changes' in Word files as these are lost in PDF conversion.

I should be grateful if you would also provide a point-by-point response detailing how you have dealt with the points raised by the reviewers in the 'Response to Reviewers' box. Please attend to all of the reviewers' comments. If you do not agree with any of their criticisms or suggestions please explain clearly why this is so.

First revision

Author response to reviewers' comments

We would like to thank the referees for their thoughtful comments on our manuscript. We appreciate that both reviewers pointed out the quality of our work, as well as the significance and impact of the findings. Our point-by-point responses to the reviewers´ comments are highlighted in blue. Text changes in the manuscript are marked in red.

Reviewer #1 (Evidence, reproducibility and clarity (Required)):

The epithelial diffusion barrier in triangular junctions is initially formed by a protein complex of Aka, Gli and M6. Aka and M6 act upstream of Gli. GPM6a, the vertebrate homolog of M6 is palmitoylated, whose functional implications have not thoroughly been analyzed, yet. It order to better define the function of M6 and especially the role of the palmitoyl moity, the authors conducted a genetic analysis of M6 in Drosophila embryos.

They first establish a genetic system with defined mutants (deletion and CS mutants which are not palmitoylated), tagged protein at the genetic locus and an quantitative assay for protein enrichment at triangular junctions. Secondly they provide biochemical evidence that M6 is palmitoylated at a cluster of three conserved cysteine residues in vivo. With a palmitoylation-deficient mutant, thirdly the authors investigate the function of the palmitoyl moiety for protein

© 2024. Published by The Company of Biologists under the terms of the Creative Commons Attribution License (https://creativecommons.org/licenses/by/4.0/).

localization at triangular junctions and complex formation with the other proteins at triangular junctions.
The authors reveal a quantitative function of the palmitoyl moiety at triangular junctions with respect to enrichment and initial accumulation but not for later functions during growth of triangular junctions. The lower enrichment of the non-palmitoylated M6 mutant are sufficient for recruitment of Aka and Gli. Importantly, reducing Aka in combination with the non- palmitoylated mutant leads to a strong phenotype with respect to Gli localization and and leads to a genetically synthetic embryonic lethality. Fourthly, on a molecular level, the palmitoyl residue mediates binding to Aka but is not required for di/oligomerization of M6 itself as shown by immunoprecipitation from embryonic lysates.

Though the function of M6 acting together with Aka and Gli has been demonstrated previously, molecular details of the interactions and targeting of the proteins to triangular junctions have remained unclear. Similarly, although palmitoylation of the vertebrate homologue has been previously demonstrated, its functional implications have not been investigated in a physiological context with stringent genetics. The current study provides convincing data about the role of the palmitoylated moiety of M6. Importantly, the authors manage to differentiate a function of the palmitoyl residue in initial accumulation of M6 at triangular junctions versus maintenance. Also the authors manage to reveal an essential function of M6 palmitoylation when the dose of Aka is reduced. In summary, the study provides novel and interesting insights into the detailed molecular requirements of epithelial barrier formation. Although the quality of the data and analysis provides an argument for publication on its own, it may be noted that similar mechanisms may underlie barrier formation at triangular junction in vertebrates given the conservation of the protein components.

Minor comments

L100: it is stated that "... is not detectable on other known TCJ components". What about Angulin-1, which is palmitoylated?

We apologize for the misleading sentence. The passage refers only to Drosophila TCJ proteins as shown in Figure 2C. We rephrased the sentence as follows:
"… is not detectable on other known TCJ components in Drosophila."

L122 In my understanding all M6 isoforms contain an element which is sufficient. Not "required".

Thank you for pointing out this logical mistake. We changed the statement as follows: "*We conclude that all M6 isoforms contain the element(s) sufficient for vertex localization.*"

L336. The allele designation "DeltaPalm" is misleading. A designation like "3xCS" would be more better because three defined cysteine residues are mutated.

We agree and we changed the allele designation to "*3xCS*", as suggested by the reviewer.

L329 A reference to FLYBASE is missing. Similarly not reference to stock centers are provides. To document the importance of the community services it is essential that their services are properly cited in a way that can be automatically tracked, e. g. by a literature citation.

We appreciate the comment, and we agree about the importance of properly acknowledging community services. We added a reference to FlyBase (Thurmond *et al.* (2019) Nucleic Acids Research 47, D759-65) and the following statement to the acknowledgement section:

"*We acknowledge service by the Developmental Studies Hybridoma Bank, created by the NICHD of the NIH and maintained at the University of Iowa, and by the Bloomington Drosophila Stock Center (NIH P40OD018537).*"

© 2024. Published by The Company of Biologists under the terms of the Creative Commons Attribution License (https://creativecommons.org/licenses/by/4.0/).

Reviewer #2 (Evidence, reproducibility and clarity (Required)):

Summary:

This article by Raphael Schleutker and Stefan Luschnig addresses the importance of S-palmitoylation of the proteolipid protein M6, one of the three components of tricellular septate junction along with Anakonda and Giotactin, in the assembly of tricellular junctions using Drosophila embryo as a model system. Using a combination of state-of-the-art genome engineering, live imaging and biochemistry, the authors demonstrated that M6 is palmitoylated in vivo, elegantly identified the cysteine residue that is palmitoylated, showed that this modification is essential for interaction with Anakonda and provided convincing evidence that palmytoylation is required for the initial assembly of tricellular junctions.

Major comments

- Are the claims and the conclusions supported by the data or do they require additional experiments or analyses to support them?

The claims are largely supported by the very high quality data assembled in this article. I have just a few concerns that can be resolved by modifying the text or, optionally, by carrying out additional experiments:
1- in the summary (lane 40) and all along the article it is stated that 'Abolishing M6 palmitoylation leads to delayed accumulation of M6 and Aka at vertices but does not affect the rate of TCJ growth or mobility of M6 or Aka.' However, whilst the data presented convincingly demonstrate the delayed localization of GFP::M6 delta Palm at TCJ, that of Aka at TCJ is not shown. Although I think this is a reasonable hypothesis, without showing Aka localization, this claim is too strong and should be toned down, or better (optional) show the dynamics of Aka localization.

We agree with the reviewer. We showed that Aka is able to localize at TCJs in the absence of M6 palmitoylation (Fig. 3N, P), but we did not provide data on the dynamics of Aka localization. We have now added new data (time-lapse movies analogous to those shown for GFP::M6 in Fig. 4) to analyze the dynamics of Aka::GFP localization in control and in *M6*^{3xCS} embryos. As observed for GFP::M6, accumulation of Aka::GFP at vertices is delayed in the absence of M6 palmitoylation, whereas the rate extension of Aka::GFP along vertices after initial accumulation is not significantly altered. We show these new results in Supplemental Figure 3 and describe them in the revised manuscript (line 203): "*A similar delay in initial vertex accumulation, but not in subsequent extension along vertices, was observed for Aka::GFP in M6*^{3xCS} *mutant embryos (Fig. S3). These findings suggest that efficient initial accumulation of Aka and M6 at vertices depends on M6 palmitoylation, while the subsequent extension of TCJ complexes during TCJ growth is independent of M6 palmitoylation.*".

Lanes 184 and 197 'indicating that effcient TCJ formation depends on M6 palmitoylation.' TCJ formation is not assessed here, what is measured is the localization of M6 at vertex. I suggest to amend the text accordingly.

We agree that the presented results address the localization of M6 rather than the process of TCJ formation. We therefore rephrased the corresponding statements as follows:

L184: "*Thus, although palmitoylation is not essential for vertex localization of M6, it promotes transport to or maintenance of M6 at vertices.*"

L197: "*... indicating that efficient TCJ localization of M6 depends on palmitoylation.*"

2- Fig. 5C and lane 292' Lack of M6 palmitoylation reduces, although it does not completely abolish, the interaction with Aka, ' In Fig. 5C, GFP::M6 efficiently co-precipitates three forms of Aka with different molecular weights. The two upper bands are highly enriched in the GFP::M6 coIp. In contrast, GFP::M6 delta Palm seems to coIp only the low molecular weight form of Aka. Could the

© 2024. Published by The Company of Biologists under the terms of the Creative Commons Attribution License (https://creativecommons.org/licenses/by/4.0/).

authors explain what the three forms of Aka are, and provide potential explanations or interpretations of this result?

We appreciate the comment. The ectodomain of Aka protein is proteolytically cleaved, as we showed previously (Byri *et al.* Dev Cell 2015). Antisera raised against a peptide in Aka´s cytoplasmic domain detect C-terminal fragments of approximately 140 and 160 kDa, respectively, which correspond to the upper two bands that co-precipitate with GFP::M6. While these two bands are always detectable, the occurrence of the third, lower molecular weight band of approx. 130 kDa that co-precipitates with palmitoylation-deficient GFP::M6(3CS) turned out to be variable.

We re-examined the specificity of the 130 kDa band by probing lysates from wild-type or Aka-deficient (*aka*^{L200}) embryos, as well as lysates from S2R+ cells transfected with an *aka* cDNA expression construct or from untransfected control cells (see Fig. 1 below). This revealed that the 140 and 160 kDa bands are specifically detected by the anti-Aka antiserum, whereas the 130 kDa band is detected also in lysates of Aka-deficient embryos or from untransfected S2R+ cells, indicating that this band is bound non-specifically by the antiserum, as is a smaller band of 50 kDa in embryo lysates. We added a star to the corresponding panels in Figure 5 to mark the positions of non-specific bands.

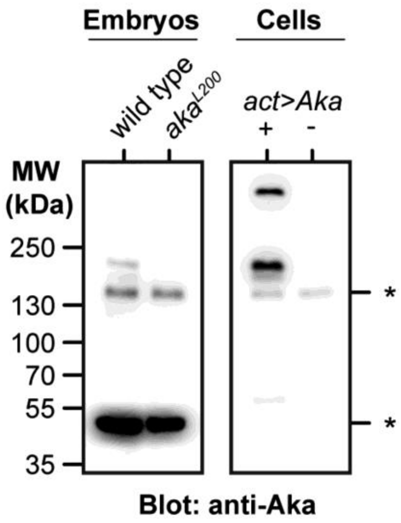

Fig.1: Immunoblots of lysates from Aka-expressing and Aka- deficient embryos or S2R+ cells probed with anti-Aka antiserum.

- Please request additional experiments only if they are essential for the conclusions. Alternatively, ask the authors to qualify their claims as preliminary or speculative, or to remove them altogether.

- Are the data and the methods presented in such a way that they can be reproduced?

The data are of very high quality and the methods sufficiently described (with appropriate references where necessary) and presented in such a way that they can be reproduced.

- Are the experiments adequately replicated and statistical analysis adequate?

Although the microscopy data are perfectly quantified and the appropriate statistical tests are used, unless I am mistaken, the number of replicates and the number of independent experiments carried out in biochemistry (Fig. 2 and Fig. 5) are not indicated.

We amended the legends of Fig. 2 and Fig. 5 to indicate the number of independent repeats for the biochemistry experiments. Two independent repeats were carried out for each acyl- biotin-exchange experiment shown in Figure 2B, C,F. At least three independent experiments were

© 2024. Published by The Company of Biologists under the terms of the Creative Commons Attribution License (https://creativecommons.org/licenses/by/4.0/).

performed for each IP experiment shown in Figure 5.

Minor comments:

- Specific experimental issues that are easily addressable.

1- The localization of M6 on living specimens, GFP::M6 enriched at the tricellular junction, differs from the localization of M6 detected by anti-M6 on fixed samples, i.e. M6 homogenous distributed at the bicellular junction, no enrichment at the tricellular junction. Please comment and possibly explain the reason for the difference in localisation. Is the anti-GFP staining on the GFP::M6 sample restricted to the bicellular junction without apparent TCJ enrichment?

As noted by the reviewer, the distribution of M6 protein appears to differ between living and fixed specimens. While we have no experimentally validated explanation for this observation, we speculate that vertex-localized M6 protein may not be efficiently recognized by the anti-M6 antiserum, possibly because the epitope may be inaccessible on M6 protein that is incorporated into the TCJ protein complex. The difference does not seem to result from the fixation and staining procedure, as staining with anti-GFP antibodies reproduces the distribution of GFP::M6 seen in live embryos. We added close-up images of epidermal cells to Fig. S1 to illustrate this point.

2- In Fig. S2, isoforms E and F are expressed at low level but fully rescue Gli localization but not Aka. These results are somewhat surprising if Gli localization relies on Aka and M6 localization at TCJ. Is localization of M6 at TCJ important or is it the expression of M6 that matters? Would it be possible to compare the expression levels of the different isoforms?.

Thank you for the thoughtful comment. We agree with the reviewer´s suggestion that the levels of M6 protein are likely to play an important role here. TCJ localization of M6 does not appear to be critical when overall levels of M6 at the plasma membrane are sufficiently high, as in the overexpression experiment referred to here (Fig. S2). M6 isoforms E and F show substantially lower expression levels (see below) compared to all other M6 isoforms, and do not fully rescue Aka localization. Albeit surprising, these results suggest that TCJ localization of Aka depends more strongly than that of Gli on the levels of M6. To substantiate this point, we quantified the levels of each GFP-tagged M6 isoform at the plasma membrane (see below).

3- lane 118 'Notably, vertex enrichment varied between M6-GFP isoforms and was inversely proportional to overall signal intensity, suggesting that saturation effects upon overexpression impede vertex enrichment. Consistent with this notion, endogenous GFP::M6CA06602 showed higher vertex enrichment (7.8-fold; Fig. 1E, L) than the individual overexpressed isoforms.' To conclude that all isoforms contain the elements for vertex localization, it would be interesting to provide the level of expression (signal intensity) for all M6 isoform as well as M6deltaPAlm-GFP to appreciate the threshold above which saturation is achieved? Or better (optional) to express the different isoforms in a M6 mutant background. Could the authors exclude the possibility that the position of the GFP moiety affect the localization of M6 at TCJ?

As suggested by the reviewer, we analyzed the expression levels (GFP signal intensity at the plasma membrane) of all individual GFP-tagged M6 isoforms as well as of palmitoylation- deficient M6(3xCS)-GFP in epidermal cells. For each construct, we compared the overall signal intensity to the enrichment factor at TCJs, which revealed an inverse relationship (see below).

© 2024. Published by The Company of Biologists under the terms of the Creative Commons Attribution License (https://creativecommons.org/licenses/by/4.0/).

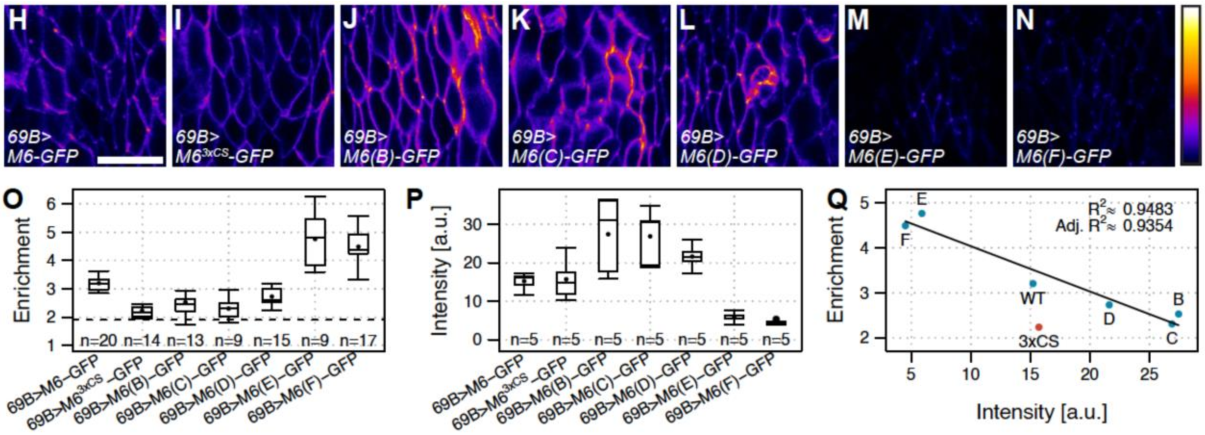

Importantly, this analysis also revealed that wild-type M6-GFP and palmitoylation-deficient M6-GFP(3xCS) are expressed at comparable levels, indicating that reduced vertex enrichment of M6-GFP(3xCS) is not a consequence of altered protein levels. We added these new data to Figure S2 in the revised manuscript and refer to them in the text (line 117).

The reviewer also raised the possibility that the position of the GFP tag may affect the localization of M6 at TCJs. We would like to note that in the overexpressed UAS-M6-GFP constructs the GFP tag is always fused to the M6 C-terminus, which is invariant between M6 isoforms. Furthermore, we showed that both N- and C-terminally GFP-tagged M6 variants localize to TCJs, excluding the possibility that the position of the GFP tag has a major influence on the localization of GFP-M6 fusion proteins.

4- lanes 187 '...in a single spot that subsequently extends basally with a speed of 0.09 µm/min ' The images are presumably projections along the apical basal axis, so it is difficult to appreciate the apical to basal extension, perhaps an orthogonal section would help.

We appreciate the reviewer´s point that apical-to-basal extension of GFP::M6 accumulation along vertices is difficult to see in the images in Fig. 4A',B'. Due to the oblique prismatic shape of the epidermal cells seen here in an *en-face* view, vertices are tilted. We tried to generate orthogonal sections, but the resolution in Z of the time-lapse images was not sufficient for that. To clarify the point, we added a new time-lapse movie (acquired with higher spatial and temporal resolution and including orthogonal projections) of GFP::M6 accumulation along a single vertex (Video S1). In this context, we would like to refer to our previous work (Wittek et al. 2020), where we show that M6 protein initially accumulates in a spot at the apical tip of the vertex and that this accumulation subsequently extends basalwards along the vertex.

- Are prior studies referenced appropriately?

yes

- Are the text and figures clear and accurate?

yes

Reviewer #2 (Significance (Required)):

Provide contextual information to readers (editors and researchers) about the novelty of the study, its value for the field and the communities that might be interested.

The following aspects are important:

Tricellular junctions are hot spots integrating mechanical and chemical inputs that are essential to

© 2024. Published by The Company of Biologists under the terms of the Creative Commons Attribution License (https://creativecommons.org/licenses/by/4.0/).

ensure epithelia homeostasis. It is therefore essential to understand how the components of tricellular junctions are located and assembled to form functional tricellular junctions. The authors brilliantly demonstrate the key role of S-palmitoylation in M6 localization and ability to interact with Aka in vivo. The fact that the role of palmitoylation appears to be conserved for the assembly of vertebrate TCJs, made up of components that are not conserved throughout evolution, indicates a fundamental function of palmitoylation in protein-protein interactions at the level of TCJs and in their vesicular trafficking. As palmitoylation is reversible, this work also raises the question of how palmitoylation is regulated in time and space to ensure the plasticity of TCJs in developing epithelia.

- General assessment: provide a summary of the strengths and limitations of the study. What are the strongest and most important aspects? What aspects of the study should be improved or could be developed?

- Advance: compare the study to the closest related results in the literature or highlight results reported for the first time to your knowledge; does the study extend the knowledge in the field and in which way? Describe the nature of the advance and the resulting insights (for example: conceptual, technical, clinical, mechanistic, functional,...).

This study follows on from that showing the role of Angulin1 palmitoylation in its localization to tricellular junctions in vertebrates. The present study demonstrates the conserved nature of the role of this post-translational modification in the assembly of complex membrane structures essential for epithelial homeostasis. In addition, it demonstrates the dynamic nature and temporality of the role of palmytoylation in the early stages of recruitment of M6 to the vertex, opening up numerous hypotheses for future work at the conceptual and fonctional levels, elegantly presented in the discssion.

- Audience: describe the type of audience ("specialized", "broad", "basic research", "translational/clinical", etc...) that will be interested or influenced by this research; how will this research be used by others; will it be of interest beyond the specific field?

I believe this article is dedicated to a rather broad audience. Although this article may at first appear to be aimed at specialists, the findings go beyond the interest in tricellular junctions in Drosophila, since the role of palmytoylation of tricellular components appears to be conserved in vertebrates. In addition, this study will have an impact on the overall cell biology community, including membrane trafficking and the role of lipid modification additions on the subcellular dynamics of transmembrane proteins in a physiological context.

- Please define your field of expertise with a few keywords to help the authors contextualize your point of view. Indicate if there are any parts of the paper that you do not have sufficient expertise to evaluate.

I am a developmental cell biologist, with an expertise in epithelial junctions and epithelial tissue homeostasis, using vertebrate and invertebrate model systems.

Second decision letter

MS ID#: JOCES/2023/261916

MS TITLE: Palmitoylation of proteolipid protein M6 promotes tricellular junction assembly in epithelia of Drosophila

AUTHORS: Raphael Schleutker and Stefan Luschnig
ARTICLE TYPE: Research Article

© 2024. Published by The Company of Biologists under the terms of the Creative Commons Attribution License (https://creativecommons.org/licenses/by/4.0/).

Thank you for sending your manuscript to Journal of Cell Science through Review Commons.

I am happy to tell you that your manuscript has been accepted for publication in Journal of Cell Science, pending standard ethics checks.

© 2024. Published by The Company of Biologists under the terms of the Creative Commons Attribution License (https://creativecommons.org/licenses/by/4.0/).
